# A hybridizing-enhanced differential evolution for optimization

**DOI:** 10.7717/peerj-cs.1420

**Published:** 2023-06-01

**Authors:** Mojtaba Ghasemi, Mohsen Zare, Pavel Trojovský, Amir Zahedibialvaei, Eva Trojovská

**Affiliations:** 1 Department of Electronics and Electrical Engineering, Shiraz University of Technology, Shiraz, Iran; 2 Department of Electrical Engineering, Jahrom University, Jahrom, Iran; 3 Department of Mathematics, Faculty of Science, University of Hradec Králové, Hradec Kralove, Czech Republic; 4 Department of Electrical and Computer Engineering, Tarbiat Modares University, Tehran, Iran

**Keywords:** Optimization, Differential evolution, Gray wolf optimizer, Stochastic optimization, Exploration, Exploation, Metaheuristic, Hybrid optimization, Generalized gray wolf optimization, CEC-2019 benchmark functions

## Abstract

Differential evolution (DE) belongs to the most usable optimization algorithms, presented in many improved and modern versions in recent years. Generally, the low convergence rate is the main drawback of the DE algorithm. In this article, the gray wolf optimizer (GWO) is used to accelerate the convergence rate and the final optimal results of the DE algorithm. The new resulting algorithm is called Hunting Differential Evolution (HDE). The proposed HDE algorithm deploys the convergence speed of the GWO algorithm as well as the appropriate searching capability of the DE algorithm. Furthermore, by adjusting the crossover rate and mutation probability parameters, this algorithm can be adjusted to pay closer attention to the strengths of each of these two algorithms. The HDE/current-to-rand/1 performed the best on CEC-2019 functions compared to the other eight variants of HDE. HDE/current-to-best/1 is also chosen as having superior performance to other proposed HDE compared to seven improved algorithms on CEC-2014 functions, outperforming them in 15 test functions. Furthermore, jHDE performs well by improving in 17 functions, compared with jDE on these functions. The simulations indicate that the proposed HDE algorithm can provide reliable outcomes in finding the optimal solutions with a rapid convergence rate and avoiding the local minimum compared to the original DE algorithm.

## Introduction

Evolutionary optimization algorithms are random search-based algorithms that model the biological evolution of organisms in various search processes and they are usually divided based on their inspiration sources Akyol & Alatas (2017) and Akyol (2022). The genetic algorithm (GA) is the most well-known evolutionary algorithm (Holland, 1992). Guided search techniques such as metaheuristic methods are among the intelligent optimization approaches that use the information obtained in the search process as a guide to select the appropriate solutions to solve complex problems. These methods have several advantages, such as the ability to search effectively in large spaces in a short time, no need for the existence of the gradient of an objective function, the ability to escape from local optimal solutions, low computational cost, usability for work with a vast number of decision variables for problems with large dimensions, usability for optimization in discrete or continuous space or complex optimizations, easy application and using possible rules in the search process instead of deterministic rules.

The differential evolution (DE) optimization method is the relatively powerful and widely used technique of metaheuristic optimization introduced in 1995 by Storn & Price (1997). This algorithm starts by forming a random initial population in a predetermined range. Each selection (member) is a potential solution for some optimization problem, in which the member position is improved by applying different operators in successive iterations leading to the optimal solutions. One of the main differences between GA and DE is the selection operator. In GA, the selection of parents depends on the members’ merits, while in the DE algorithm, all members have the same chance of being selected. Furthermore, unlike the GA optimization method, in DE, the mutation steps do not follow a specific probabilistic distribution. Instead, the difference between members is used to guide the search process, which effectively increases the participation of members in finding subsequent solutions. Another critical difference between GA and DE is the order in applying the mutation and crossover operators.

In DE, parents are created at the mutation stage before the crossover, while in the GA algorithm, the parents are selected from the current population and then the crossover and mutation are performed. Furthermore, the user must select a few control parameters (namely, the crossover rate $CR$, the population size ${N_{pop}}$, and the scale factor $F$) in DE, which may vary for different problems, which significantly impacts the algorithm performance. Besides, the convergence rate of the DE algorithm is relatively slower for more test functions and optimization problems, especially compared to the particle swarm optimization algorithm (PSO). Therefore, in recent years, many studies have investigated the evolution and application of some enhanced versions of the DE algorithm, briefly introduced in the following.

Brest et al. (2006) presented jDE, a DE variant with a unique characteristic in which parameters ( $CR$ and $F$) have been adjusted during every evolution while using identical mutational techniques as in the canonical DE algorithm. According to distinctive ideas developed in the algorithm jDE, better control parameters are usually related to better trial vectors and *vice versa*. A better group of control parameters yield a better group of trial vectors, which must be held in the next generation’s control parameter proliferation. This response system was the foundation for later DE variants, like jSO (Brest, Maučec & Bošković, 2017), which all won first place in optimization competitions. In addition to the mentioned algorithms, Islam et al. (2012), Yu et al. (2013), and Meng et al. (2020) suggested other hierarchical archive strategies “DE/target-to-gr best/1”, “DE/best/1”, “DE/pbest/2”, and “DE/target-to-pbest/1” based on mutations. Although all these mutation strategies achieved competitive results, in some benchmarks, they had a slow convergence rate, and population size adaptation could be improved further.

Qin, Huang & Suganthan (2009) designed the self-adaptive DE (SaDE) algorithm with numerous approaches of mutations in another class. The main idea of the algorithm SaDE is the fact that mutation approaches are dynamically changed due to the length of the period for which a strategy functioned in each generation. Wang, Cai & Zhang (2011) presented the composite DE (CoDE) algorithm with predetermined control parameters and approaches of mutation. Every mutation strategy has haphazardly consisted of a pair of control parameters in each CoDE’s generation. A two-component mechanism was introduced by Gong et al. (2011) to energetically pick the mutation approaches suggested in JADE, which achieved better results. Nevertheless, there is a crucial flaw in these DE versions of the multi-mutation strategy, which can lead to a disaster in the solution results for the wrong mutation. Gong & Cai (2013) suggested a ranking-based system in which some of the vectors in the mutation approach are comparably chosen from the population’s ranked individuals. Cai et al. (2018) suggested the method based on a social-learning to improve previous DE versions. In this algorithm, the population’s social network was developed based on the individuals’ social impact. While these frameworks primarily concentrated on DE versions with predetermined population sizes, recent findings have shown that DE variants with reduced population sizes outweigh their counterparts with predetermined population sizes. However, using a population size reduction scheme does not implicate improving some DE versions with a fixed size of the population (Meng & Yang, 2021).

Since the discovery of DE, several research projects have been conducted to study some self-adaptive techniques to change control parameters. A novel historical and heuristic DE (HHDE) has been suggested by Liu et al. (2018) in which the parameter adaptation process is based on historical heuristics. A self-adaptive DE was proposed by Fan & Yan (2015) in which the control parameters are dependent on the growth of zoning, and their combinations have varying functions in various search regions.

To dynamically change DE’s parameters, Ochoa, Castillo & Soria (2014) used fuzzy logic control systems. By dynamically adapting the mutation parameters, Ochoa, Castillo & Soria (2020) presented an improved DE algorithm for designing fuzzy controllers for the application of nonlinear plants. Additionally, a shadowing type-2 fuzzy inference technique (Castillo et al., 2019a) and a rapid interval type-2 fuzzy system strategy (Castillo et al., 2019b) were designed to modify DE’s parameters dynamically. Above that, much study has been conducted on techniques for offspring generation, such as mutation and crossover operators.

Qiu, Tan & Xu (2017) suggested, by studying the crossover operator, numerous exponential recombination for DE in which many parts of the mutant and target vectors are substituted to find the trial vector. A new DE with hybrid linkage crossover was introduced by Cai & Wang (2015), which extracts and integrates the problem linkage information to be optimized into the crossover phase. A new interactive information scheme (IIN) replaces the best solution in the “current to-best” variant with many best solutions (Zheng et al., 2018), in which using a weighted mixture of ranking information, a steering vector is constructed, and IIN is included into the mutation operator to provide hopeful guiding data. Designing new mutation techniques falls into the first group. Zhang & Sanderson (2009) suggested a “current to-p-best” approach to direct the mutation scheme, in which numerous best options substitute the best outcome in the version “current to-best.” Liu et al. (2019) addressed a DE’s adaptive tune framework for modifying the usual coordinate system, in which the crossover operator is applied to both the Eigen coordinate system and the initial coordinate systems. Finally, several enhanced mutation schemes have been studied to investigate the mutation operator and improve its exploration capability. These mutation arrangements can be divided generally into the below classes.

An enhanced fitness-adaptive differential evolution algorithm (EFADE) based on a system of triangular mutations was proposed (Mohamed & Suganthan, 2018), in which a triplet’s combinatorial convex vector is described by three haphazardly designated solutions and their difference vectors. Combining multiple mutation techniques falls into the second group. Wu et al. (2016) recommended the multi-population ensemble differential evolution method (MPEDE) based on a multiple-mutations approach with one subpopulation indicator. The third class denotes hybridization with other search approaches, like machine learning methods, swarm intelligence, and other EAs. A DE variation based on individual variability in knowledge (DI-DE) was proposed by Tian, Li & Yan (2019), in which multiple mutation methods are used and chosen based on an adaptive strategy for superior and inferior individuals. *E.g*., Cai et al. (2020) developed a new DE system called self-organizing neighborhood-based differential evolution (SON-DE), in which neighborhood connections between solutions are developed to drive the mutation process. Zhang et al. (2016) suggested a self-organizing multi-objective evolutionary algorithm (SMEA) based on learning the solutions’ neighborhood correlation using SOM and picking these neighbors to create for every answer the pairing pool.

For multimodal optimization problems, Fang, Zhou & Zhang (2018) suggested a hybrid algorithm combining DE and estimation of distribution algorithm (EDA), in which Gaussian probabilistic EDA models and the DE’s evolutionary operators were combined to generate offspring. Jadon et al. (2017) suggested a hybrid artificial bee colony with differential evolution (HABCDE), in which scout, onlooker, and working bee phases are changed based on DE’s search tools. Wang et al. (2017) suggested a DE algorithm with a dual-strategy, in which affinity-spreading clusters are used to choose various sub-populations for stating the following generation. Xin et al. (2011) thoroughly analyzed current hybrids in DE and PSO, considering five hybridization variables and developing a systematic taxonomy for hybridization approaches. Recently, an increasing range of topics has been produced on neighborhood frameworks for DE to improve the mutation operator’s searchability. The following two methods can be used to describe the neighborhood knowledge used in DE. The first technique consists of a fundamental population topology based on the neighborhood, and the neighbors of each solution in the current population are distributed by their ordinals (Cai et al., 2021). Das et al. (2009) suggested a new version of DE based on global and local neighborhoods (DEGL), in which the local and global neighborhoods are described using ring topology followed by linear arrangements using the mutation operator. For instance, Dorronsoro & Bouvry (2011) used DE to describe each solution’s neighborhood list and then selected the neighborhood mutation parents for every goal option. An improved multi-elite mutation approach was also suggested by Cui et al. (2018) for DE, in which elites are adjustably chosen from the ring topology’s neighborhood as well as the present population’s highest best solutions. Cai et al. (2017b) designed a neighborhood-guided differential evolution (NGDE) that directs the searching by DE by integrating the relationships indicated by the topological ring and the fittest merit of every response. Ge et al. (2017) offered the distributed differential evolution with an adaptive population model (DDE-AMS), which energetically allocated computing resources across several subpopulations. De Falco et al. (2017) presented the improved distributed differential evolution (DDE), in which an asynchronous adaptive algorithm is used to pick the migration’s subpopulations. The algorithm DDE showed good applicability for large-scale optimization problems. A multi-topological DE (MTDE) with topology adaptability was proposed by Sun et al. (2018) that is individual-dependent, in which individual variations in search roles are employed to select the most appropriate topology. A multi-topology DE (MTDE) proposed by Sun et al. (2018) is based on an individual-dependent topology mechanism with individual searching roles. A neighborhood-adaptive differential evolution (NaDE) was introduced by Cai et al. (2017a), in which both mobile and ring topologies are dynamically selected as the sample of previous successful and failing experiences for each solution neighborhood. Furthermore, the second method of employing DE neighborhood information is to define it based on decision/objective space population data (Cai et al., 2021). A proximity-based DE system (ProDE) was suggested by Epitropakis et al. (2011), in which the probability of picking a neighbor as a parent is inversely related to their distance from the desired solution. The fitness-and-position dependent selection (FPS-DE) was suggested by Cai et al. (2016), in which each solution’s fitness merit and location data are employed to determine everyone’s impact, and the parents are chosen for mutation based on their impact. Wang et al. (2014) introduced the multi-objective sorting-dependent mutation operators (MSDE) with a non-dominated sorting approach that sorts all solutions by their fitness and diversity influence and according to parents’ ranking merits. Another variant of the algorithm DE improved by special neighborhood and direction information (NDi-DE) was offered by Cai & Wang (2013). In NDi-DE, neighbors are designated based on probability selection using location data, and the directional data is built using the best and worst near neighbors. A species-based differential evolution method with self-adaptation strategies (self-CSDE) was offered by Gao, Yen & Liu (2013). Here, population fitness merits are employed to assess species seeds, and population location data is used to choose parents within the same species.

Different types of DE algorithms in previous studies indicate that these algorithms may only work well to solve some optimization problems and still need improvement for particular types of problems. Since one of the main problems of the DE algorithms (DEs) is the low convergence speed, this study introduces the hunting phase of grey wolfs as an auxiliary mutation vector to the DE algorithm to accelerate the convergence rate and achieve better optimal solutions. In the mutation phase of the proposed algorithm in each iteration, instead of the primary vector in the DEs, the hunting vector in Gray Wolf Optimizer (GWO) is used with the predetermined probability for each member, and then the crossover and selection operators like as DE are applied.

This article is organized as follows: The first section, “Overview of DE and GWO algorithms” introduces the conventional versions of DE and GWO algorithms. Next, a description of the proposed HDE algorithm is given in the section “The proposed HDE algorithm,” and simulation and evaluation studies on the handling of optimization tasks are presented in the section “Simulation results.” Finally, some concluding remarks are given in the section “Conclusions”.

### Overview of DE and GWO algorithms

This section, at first, briefly presents the framework of DE and GWO algorithms, and then the proposed algorithm (HDE) is introduced by combining these two algorithms.

### Differential evolution

The original version of the differential evolution algorithm was introduced by Storn & Price (1996). Das, Mullick & Suganthan (2016) has provided a comprehensive description of many applications of the DE algorithm and its improved versions, which appeared in recent years. Like other evolutionary algorithms, an initial population is generated at first. Then, some operators, including composition, mutation, and crossover, are applied to the population to form a new population. In the next step, *i.e*., the selection phase, the new population is compared to the current population based on objective function merits. Then, the best members enter the next stage as the next generation. This process continues until the desired results are achieved. This section describes the performance steps of the HDE algorithm.

From a mathematical point of view, a population can be represented using a vector of population members (*i.e*., search agents) $\left( {\vec X_1^{Iter},\vec X_2^{Iter}, \ldots ,\vec X_{{N_{pop}}}^{Iter}} \right)$, where the $i$-th population member $\vec X_i^{Iter}$ has the form $\vec X_i^{Iter} = \left( {x_{i,1}^{Iter},x_{i,2}^{Iter}, \ldots ,x_{i,D}^{Iter}} \right)$. Here, $x_{i,j}^{Iter}$ is the $j$-th dimension of the $i$-th search agent $\vec X_i^{Iter}$ (*i.e*., the $j$-th decision variable), ${N_{pop}}$ is the number of search agents in a population, and $D$ is the number of decision variables (the dimension of the problem). The indices $i$ and $Iter$ are characterized as $Iter\; = \; 1,\; 2, \ldots ,Ite{r_{max}}$ and $i\; = \; 1,\; 2, \ldots ,\; {N_{pop}},$ where $Ite{r_{max}}$ is the maximal number of iterations.

1. Initial population generation

The minimal and maximal values for the $j$-th decision variable ( $j\; = \; 1,\; 2,\; \ldots ,\; D$) of the problem are denoted by ${x_{min,j}}$ and ${x_{max,j}}$, respectively. The initial population (*i.e*., $Iter = 1$) with the total number of members of the population ${N_{pop}}$ in $D$ dimensions can be generated as Eq. (1):

(1) $$x_{i,j}^{Iter} = ran{d_j}\left( {0,1} \right)\cdot \left( {{x_{max,j}} - {x_{min,j}}} \right),$$

where $ran{d_j}\left( {0,1} \right)$ is a random function that generates a real number with a uniform probability distribution between 0 and 1 (*i.e*., $ran{d_j}\left( {0,1} \right) \in U\left[ {0,1} \right]$).

2. Mutation

In the DE algorithm, different strategies can be used for mutating and creating a new population Mininno et al. (2010) and Ghasemi et al. (2016). The mutation operator selects random vectors for each possible range in the starting population named $\vec X_r^{Iter}$ (like $\vec X_{{r_1}}^{Iter}$, ${\rm \; }\vec X_{{r_2}}^{Iter}$, $\vec X_{{r_3}}^{Iter}$, and $\vec X_{{r_4}}^{Iter}$) from starting population matrix and generates a new vector $\vec V_i^{Iter} = \left( {v_{i,1}^{Iter},v_{i,2}^{Iter}, \ldots ,v_{i,D}^{Iter}} \right)$ for the $Iter$-th iteration of the algorithm and the $i$-th member of the population based on the utilized mutation approach that creates a new population. Randomly chosen vectors $\vec X_r^{Iter}$must be dissimilar from each other. The resultant vector $\vec V_i^{Iter}$ produced by the mutation operator has one of the following configurations as presented in Eqs. (2) to (9) Mininno et al. (2010) and Ghasemi et al. (2016):

“DE/rand/1”:

(2) $$\vec V_i^{Iter} = \vec X_{{r_1}}^{Iter} + F\cdot \left( {\vec X_{{r_2}}^{Iter} - \vec X_{{r_3}}^{Iter}} \right),$$

“DE/best/1”:

(3) $$\vec V_i^{Iter} = \vec X_{best}^{Iter} + F\cdot \left( {\vec X_{{r_1}}^{Iter} - \vec X_{{r_2}}^{Iter}} \right),$$

“DE/current-to-best/1”:

(4) $$\vec V_i^{Iter} = \vec X_i^{Iter} + F\cdot \left( {\vec X_{best}^{Iter} - \vec X_i^{Iter}} \right) + F\cdot \left( {\vec X_{{r_1}}^{Iter} - \vec X_{{r_2}}^{Iter}} \right),$$

“DE/rand/2”:

(5) $$\vec V_i^{Iter} = \vec X_{{r_1}}^{Iter} + F\cdot \left( {\vec X_{{r_2}}^{Iter} - \vec X_{{r_3}}^{Iter}} \right) + F\cdot \left( {\vec X_{{r_4}}^{Iter} - \vec X_{{r_5}}^{Iter}} \right),$$

“DE/best/2”:

(6) $$\vec V_i^{Iter} = \vec X_{best}^{Iter} + F\cdot \left( {\vec X_{{r_1}}^{Iter} - \vec X_{{r_2}}^{Iter}} \right) + F\cdot \left( {\vec X_{{r_3}}^{Iter} - \vec X_{{r_4}}^{Iter}} \right),$$

“DE/rand-to-best/1”:

(7) $$\vec V_i^{Iter} = \vec X_{{r_1}}^{Iter} + F\cdot \left( {\vec X_{best}^{Iter} - \vec X_{{r_1}}^{Iter}} \right) + F\cdot \left( {\vec X_{{r_2}}^{Iter} - \vec X_{{r_3}}^{Iter}} \right),$$

“DE/rand-to-best/2”:

(8) $$\vec V_i^{Iter} = \vec X_{{r_1}}^{Iter} + F\cdot \left( {\vec X_{best}^{Iter} - \vec X_{{r_1}}^{Iter}} \right) + F\cdot \left( {\vec X_{{r_2}}^{Iter} - \vec Y_{{r_3}}^{Iter}} \right) + F\cdot \left( {\vec X_{{r_4}}^{Iter} - \vec X_{{r_5}}^{Iter}} \right),$$

“DE/current-to-rand/1”:

(9) $$\vec V_i^{Iter} = \vec X_i^{Iter} + rand\left( {0,1} \right)\cdot \left( {\vec X_{{r_1}}^{Iter} - \vec X_i^{Iter}} \right) + F\cdot rand\left( {0,1} \right)\cdot \left( {\vec X_{{r_2}}^{Iter} - \vec X_{{r_3}}^{Iter}} \right),$$

where $rand\left( {0,1} \right) \in U\left[ {0,1} \right]$. The mutation operator has a parameter $F$, which is uniformly randomly picked in the range from 0 to 2 (*i.e*., $F \in U\left[ {0,2} \right]$), and $\left( {\vec X_{{r_1}}^{Iter} - \vec X_{{r_2}}^{Iter}} \right)$, $\left( {\vec X_{{r_2}}^{Iter} - \vec X_{{r_3}}^{Iter}} \right),$ and $\left( {\vec X_{{r_4}}^{Iter} - \vec X_{{r_5}}^{Iter}} \right)$ are diverse vectors that modify the base vector. Population members are recognized by $\vec X_r^{Iter}$, and the best individual vector with the best fitness merit in the present population at iteration $Iter$ is denoted by $\vec X_{best}^{Iter}$.

3. Crossover

This operator increases the algorithm’s strength and escapes from local optimal solutions. The crossover operation, with the constant of crossover $CR$ ( $0\; < CR < 1$), is performed for each $j$-th decision variable of the $i$-th search agent of the population at iteration $Iter$, to compute the trial vector $\vec U_i^{Iter} = \left( {u_{i,1}^{Iter},u_{i,2}^{Iter}, \ldots ,u_{i,D}^{Iter}} \right)$ as Eq. (10):

(10) $$u_{i,j}^{Iter} = \left\{ {\matrix{ {v_{i,j}^{Iter},} & {ran{d_{i,j}}\left( {0,1} \right) \le CR;}  \cr {x_{i,j}^{Iter},} & {{\rm otherwise},} \cr } } \right.$$

where $ran{d_{i,j}}\left( {0,1} \right) \in U\left[ {0,1} \right]$, $i = 1,2, \ldots ,{N_{pop}}$, and $j = 1,2, \ldots ,D.$

4. Selection

In this step, the fitness function of a new population $\vec U_i^{Iter}$, *i.e*., $f\left( {\vec U_i^{Iter}} \right)$ is evaluated and compared with the fitness of the current position $f\left( {\vec X_i^{Iter}} \right)$, and if it has a better fitness function value, it replaces the current solution, otherwise, the $i$-th member keeps its current position as Eq. (11):

(11) $$\vec X_i^{Iter + 1} = \left\{ {\matrix{ {\vec U_i^{Iter},} & {f\left( {\vec U_i^{Iter}} \right) \le f\left( {\vec X_i^{Iter}} \right);} \cr {\vec X_i^{Iter},} & {{\rm otherwise}.} \cr } } \right.$$

### GWO

This section presents the mathematical modeling of GWO (Mirjalili, Mirjalili & Lewis, 2014).

1. Social hierarchy

When developing GWO, the best solution based on the fitness function is considered as *α* wolf (the dominant wolf). Similarly, the second and third fittest solutions are denoted as $\beta$ and $\delta$ wolves. All the remaining candidates are denoted as *ω* wolves, which are directed by $\alpha$, $\beta$, and $\delta$ to pursue the hunting process.

2. Encircling prey

Eqs. (12) and (13) are proposed to mathematically model the encircling action of grey wolves.

(12) $$\vec D_\; ^{Iter} = \left| {\vec C{\rm \,*\,}\vec X_{\; p}^{Iter} - \vec X_\; ^{Iter}} \right|,$$

(13) $$\vec X_\; ^{Iter + 1} = \vec X_{\; p}^{Iter} - \vec A{\rm \,*\,}\vec D_\; ^{Iter},$$

where $Iter$ denotes the present iteration, $\vec A$ and $\vec C$ represent coefficient vectors, $\vec X_{\; p}^{Iter}$ indicates the prey’s location vector in the iteration $Iter$, and vectors $\vec X_\; ^{Iter}$ and $\; \vec X_\; ^{Iter + 1}$ are gray wolf position vectors in the iteration $Iter$ and $Iter + 1$, respectively. The parameters $\vec A$ and $\vec C$ are computed by Eqs. (14) and (15).

(14) $$\vec A = 2\vec a*\overrightarrow {{r_1}} - \vec a, {\rm where}{\rm \; }\vec a = 2\left( {1 - \displaystyle{{Iter} \over {Ite{r_{max}}}}} \right),$$

(15) $$\vec C = 2\overrightarrow {{r_2}},$$

where the components of $\vec a$ are linearly decreasing from 2 to 0, concerning the growth of the number of iterations, and $\overrightarrow {{r_1}}$, $\overrightarrow {{r_2}}$ are uniform random vectors with components in the interval [0, 1].

3. Hunting

A pack of gray wolves can detect the presence of prey and surround and enclose it. The alpha is generally in charge of the hunt. It is likely that the beta and delta sometimes also involve in hunting. In an abstract solution area, however, we do not know where the optimum prey is located. In order to computationally recreate grey wolf hunting behavior, we assume that the alpha, beta, and delta wolves contain more information about possible prey locations. As a result, the first three best solutions found so far are kept, and the other search representatives (containing the omegas) update their locations in accordance with the best search agents’ placements. The following Eqs. (16) to (18) are proposed in this regard for $i = 1,2, \ldots ,{N_{pop}}$.

(16) $$\matrix{ {\vec D_\alpha ^{Iter} = \left| {\overrightarrow {{C_1}} *\overrightarrow {{X_\alpha }} - \vec X_i^{Iter}} \right|,} \cr {\vec D_\beta ^{Iter} = \left| {\overrightarrow {{C_2}} *\overrightarrow {{X_\beta }} - \vec X_i^{Iter}} \right|,} \cr {\vec D_\delta ^{Iter} = \left| {\overrightarrow {{C_3}} *\overrightarrow {{Y_\delta }} - \vec X_i^{Iter}} \right|,} \cr }$$

(17) $$\matrix{ {\overrightarrow {{X_{\rm {\rm A}}}} = \overrightarrow {{X_\alpha }} - \overrightarrow {{A_1}} *\vec D_\alpha ^{Iter},} \cr {\overrightarrow {{X_{\rm {\rm B}}}} = \overrightarrow {{X_\beta }} - \overrightarrow {{A_2}} *\vec D_\beta ^{Iter},} \cr {{\rm \; }\overrightarrow {{X_{\rm \Delta}}} = \overrightarrow {{X_\delta }} - \overrightarrow {{A_3}} *\vec D_\delta ^{Iter},} \cr }$$

(18) $$\vec X_i^{Iter + 1} = \left( {\overrightarrow {{X_{\rm {\rm A}}}} + \overrightarrow {{X_{\rm {\rm B}}}} + \overrightarrow {{X_{\rm \Delta}}} } \right)/3$$

## The proposed hde algorithm

### Greedy non-hierarchical GWO

During the update mechanism of the algorithm GWO, the best three options are steadily saved and denoted as wolves ( $\alpha$, $\beta$, and $\delta$) and these population members lead the other members in their upgrade calculation, similarly as it works in the natural social hierarchy of grey wolf packs. It was shown the GWO algorithm converges quickly to the optimal solution for most of the well-known benchmark functions. On the other hand, this mechanism has a crucial disadvantage in optimizing some real-world problems, *i.e*., the algorithm converges rapidly but to a locally optimal solution. To overcome these drawbacks in the GWO, the G-NHGWO algorithm (Akbari, Rahimnejad & Gadsden, 2021), the best personal optimal solution $\vec X_{i,best}^{Iter}$ for the $i$-th grey wolf, $i = 1,2, \ldots ,{N_{pop}},$ has been established and stored, like in the PSO algorithm. Then, three members, ${r_1}$, ${r_2}$, and ${r_3}$, with individual best positions, respectively $\vec X_{{r_1},best}^{Iter}$, $\vec X_{{r_2},best}^{Iter}$, and $\vec X_{{r_3},best}^{Iter}$, are selected randomly and utilized to lead the population to update the new positions as Eqs. (19) to (21):

(19) $$\matrix{ {\vec D_{{r_1}}^{Iter} = \left| {{{\vec C}_1}*\vec X_{{r_1},best}^{Iter} - \vec X_{best}^{Iter}} \right|,} \cr {\vec D_{{r_2}}^{Iter} = \left| {{{\vec C}_2}*\vec X_{{r_2},best}^{Iter} - \vec X_{best}^{Iter}} \right|} \cr {\vec D_{{r_3}}^{Iter} = \left| {{{\vec C}_3}{\rm *\; }\vec X_{{r_3},best}^{Iter} - \vec X_{best}^{Iter}} \right|,} \cr }$$

(20) $$\matrix{ {{{\vec X}_{{r_1}}} = \vec X_{{r_1},best}^{Iter} - {{\vec A}_1}*\vec D_{{r_1}}^{Iter},} \cr {{{\vec X}_{{r_2}}} = \vec X_{{r_2},best}^{Iter} - {{\vec A}_2}*\vec D_{{r_2}}^{Iter},} \cr {{\rm \; }{{\vec X}_{{r_3}}} = \vec X_{{r_3},best}^{Iter} - {{\vec A}_3}*\vec D_{{r_3}}^{Iter},} \cr }$$

(21) $$\vec X_i^{Iter + 1} = \left( {{{\vec X}_{{r_1}}} + {{\vec X}_{{r_2}}} + {{\vec X}_{{r_3}}}} \right)/3.$$

### HDE algorithm

In the GWO algorithm, $\alpha$, $\beta$, and $\delta$ wolves always participate in the hunting phase. Therefore, using the positions of the best wolves in the hunting phase increases the quality of the exploration and accelerates the convergence of the GWO method (Akbari, Rahimnejad & Gadsden, 2021). Since one of the main problems of the DE is the low convergence speed, in this study, the hunting phase of GWO is proposed as an auxiliary mutation vector to the DE to accelerate the convergence rate and achieve more optimal solutions. In the process of the proposed HDE algorithm, in each iteration, instead of the general vector in the DE algorithm, the GWO’s hunting vector is used as the mutation vector with the probability of *H*_*m*_ for each member. Then the crossover and selection operators are applied.

In the proposed HDE algorithm, instead of using $\vec X_{{r_1},best}^{Iter}$, $\vec X_{{r_2},best}^{Iter}$, and ${\rm \; }\vec X_{{r_3},best}^{Iter}$ in (19) and (20), three members of $\overrightarrow {{X_\alpha }}$, $\overrightarrow {{\rm \; }{X_\beta }}$, and $\overrightarrow {{\rm \; }{X_\delta }}$ are used. Therefore, the new position of the $i$-th member, $i = 1,2, \ldots ,{N_{pop}}$, can be calculated using Eqs. (22) to (24).

(22) $$\matrix{ {\vec D_1^{Iter} = \left| {\overrightarrow {{C_1}} {\rm *}\overrightarrow {{X_\alpha }} - \vec X_{best}^{Iter}} \right|,} \cr {\vec D_2^{Iter} = \left| {\overrightarrow {{C_2}} *\overrightarrow {{X_\beta }} - \vec X_{best}^{Iter}} \right|} \cr {\vec D_3^{Iter} = \left| {\overrightarrow {{C_3}} *\overrightarrow {{X_\delta }} - \vec X_{best}^{Iter}} \right|,} \cr }$$

(23) $$\matrix{ {\overrightarrow {{X_{\rm {\rm A}}}} = \overrightarrow {{X_\alpha }} - \overrightarrow {{A_1}} *\vec D_1^{Iter},} \cr {\overrightarrow {{X_{\rm {\rm B}}}} = \overrightarrow {{X_\beta }} - \overrightarrow {{A_2}} *\vec D_2^{Iter},} \cr {{\rm \; }\overrightarrow {{X_{\rm \Delta}}} = \overrightarrow {{X_\delta }} - \overrightarrow {{A_3}} *\vec D_3^{Iter},} \cr }$$

(24) $$\vec X_i^{Iter + 1} = \left( {\overrightarrow {{X_{\rm {\rm A}}}} + \overrightarrow {{X_{\rm {\rm B}}}} + \overrightarrow {{X_{\rm \Delta}}} } \right)/3.$$

where the operation “ $*$” represents the Hadamard product of two vectors (*i.e*., the multiplication of vectors by multiplying their corresponding components), and the absolute value function $\left| \cdot \right|$ is calculated sequentially by the components of the vectors.

Afterward, $\vec X_i^{Iter + 1}$ is compared with $\vec X_{best}^{Iter},$ and if it has a better objective function value, it will be considered the new personal best position (selection phase). This process is repeated until reaching the maximum number of fitness evaluations $NF{E_{max}}$ (*i.e*., $NF{E_{max}}\; = \; Ite{r_{max}}\cdot {N_{pop}}$).

The HDE algorithm can be written in the following pseudocode steps:

**Step 0:** Input the optimization problem information and values of parameters ${N_{pop}}$, $D$, $NF{E_{max}}$, $F$, ${H_m}$, and $CR$.

**Step 1:** Initialization of the population of search agents (grey wolfs), we set $Iter = 1$: $\vec X_{i\; }^{Iter}$, $\; i\; = \; 1,\; 2,\; \ldots ,\; {N_{pop}}$.

**Step 2:** Compute the fitness value for all search agents.

**Step 3:** Initialize parameters $\vec a$, $\vec A$, and $\vec C$ by (14) and (15).

**Step 4: While** $Iter\cdot {N_{pop}} < NF{E_{max}}$ **Do**

**Step 5:**       Evaluate the search agents $\overrightarrow {{X_\alpha }}$, ${\rm \; }\overrightarrow {{X_\beta }}$, and $\overrightarrow {{\rm \; }{X_\delta }}$ ( $\alpha ,\; \beta ,$ and $\delta$ wolves).

**Step 6:       If** $rand < {\rm \; }{H_m}$ **Then** perform the hunting mutation using (22) to (24).

**Step 7:       Else** Perform the original mutation with a probability 1 – ${\rm \; }{H_m}$ using (2) to (9).

**Step 8:**       Update parameters $\vec a$, $\vec A$, and $\vec C$ by (14) and (15).

**Step 9:**       Evaluate all search agents’ fitness values.

**Step 10:**      Update the value of $\overrightarrow {{X_\alpha }}$, ${\rm \; }\overrightarrow {{X_\beta }}$, and $\overrightarrow {{\rm \; }{X_\delta }} .$

**Step 11:**      *Crossover operation* by (10).

**Step 12:**      Evaluation of the fitness value of the population of all search agents.

**Step 13:**      *Selection* by (11).

**Step 14:** $Iter = Iter + 1$

**Step 15: End While**.

### The computational complexity of HDE

It is essential to realize that three processes—initialization, fitness evaluation, and updating of the population—largely determine the HDE's computational complexity. First, the initialization process has a computational complexity equal to $O({N_{pop}}$) for ${N_{pop}}$ individuals. During searching for the optimal location and updating the location vector of the entire population, the updating mechanism has a computational complexity equal to $O\left( {Ite{r_{max}}\; \cdot \; {N_{pop}}} \right) + O\left( {Ite{r_{max}}\; \cdot \; {N_{pop}}\; \cdot D} \right)$, where $Ite{r_{max}}$ is the maximal number of iterations and $D$ is the dimension of the problem. Finally, HDE has a total computational complexity of $O\left( {{N_{pop}}\; \cdot \left( {\; Ite{r_{max}}\; + \; Ite{r_{max}}\; \cdot D\; + \; 1} \right)} \right).$

## Simulation results

### Experimental design

This section investigates the effectiveness and robustness of the proposed HDE algorithm on two standard benchmarks, including the CEC-2014 suite (Liang, Qu & Suganthan, 2013) and the CEC-2019 suite (Liang et al., 2019). Characteristics of the CEC-2014 suit of benchmark functions have been shown in Table 1. The CEC-2014 benchmarks have three unimodal functions ( ${F_1}$– ${F_3}$), thirteen simple multimodal functions ( ${F_4}$– ${F_{16}}$), six hybrid functions ( ${F_{17}}$– ${F_{22}}$), and eight composition functions ( ${F_{23}}$– ${F_{30}}$). The search range for each function is $\left[ { - 100,\; 100} \right],$ while the minimum value for each function equals $100\,k$, where “ $k$” is the number of the benchmark function. The characteristics of CEC-2019 are defined in Table 2. In this scenario, different HDE algorithms are compared to clarify how different strategies can affect optimization performance. In contrast, the results of CEC-2014 benchmarks are compared with some other optimization algorithms. All experiments were conducted using MATLAB 2018a on a Windows 10 computer with a 2.2 GHz Core i7 processor and 16 GB of RAM.

**Table 1:** Characteristics of the CEC-2014 suit of benchmark functions.

Number	Function type	${[{X_{min}},\; {X_{max}}]^D}$
F1	Unimodal (UF)	${\left[ { - 100,\; 100} \right]^D}$
F2		
F3		
F4	Simple multimodal (SMF)	
F5		
F6		
F7		
F8		
F9		
F10		
F11		
F12		
F13		
F14		
F15		
F16		
F17	Hybrid (HF)	
F18		
F19		
F20		
F21		
F22		
F23	Composition (CF)	
F24		
F25		
F26		
F27		
F28		
F29		
F30		

**Table 2:** Characteristics of the CEC-2019 suit of benchmark functions.

No.	Functions	$f\left( {{{\bf X}^{^*}}} \right)$	$D$	Search range
1	Storn’s Chebyshev polynomial fitting problem	1	9	[−8192, 8192]
2	Inverse Hilbert matrix problem	1	16	[−16384, 16384]
3	Lennard-Jones minimum energy cluster	1	18	[−4, 4]
4	Rastrigin’s function	1	10	[−100, 100]
5	Griewangk’s function	1	10	[−100, 100]
6	Weierstrass function	1	10	[−100, 100]
7	Modified Schwefel’s function	1	10	[−100, 100]
8	Expanded Schaffer’s F6 function	1	10	[−100, 100]
9	Happy cat function	1	10	[−100, 100]
10	Ackley function	1	10	[−100, 100]

### Application of HDE for the CEC-2014 and CEC-2019 suit of benchmark functions

In this step of optimization, we require sophisticated functions to verify the capacity of the DE algorithms to optimize real functions, such as economic load dispatch, compared to other algorithms. Therefore, we chose the CEC-2014 test functions, which have already been applied in many previous articles. Here we have utilized the number of dimensions equal to 30, the number of function evaluations of 300,000, and the population's range of all algorithms was set to 30. The parameter $a$ decreases from 2 to 0. Each optimization method was run 30 times for each function. The obtained results were used to calculate the mean value, standard deviation, and a specified convergence graph. The set of control parameters of the algorithms is shown in Table 3. However, it should be noted that the value of the control parameter $F$ is not mentioned in this table since it is the same for all algorithms and is equal to $(0.1\; + \; 0.8\; {\rm rand}())$.

**Table 3:** Selected parameters for DE and HDE methods in this article.

Algorithm	*H*_*m*_	*CR*
HDE/rand-to-best/2	0.5	0.95
DE/rand-to-best/2	–	0.95
HDE/current-to-best/1	0.9	0.9
DE/current-to-best/1	–	0.9
HDE/rand-to-best/1	0.9	0.9
DE/rand-to-best/1	–	0.9
HDE/current-to-rand/1	0.5	0.9
DE/current-to-rand/1	–	0.9
HDE/best/2	0.1	0.9
DE/best/2	–	0.9
HDE/best/1	0.9	0.9
DE/best/1	–	0.9
HDE/rand/2	0.1	0.9
DE/rand/2	–	0.9
HDE/rand/1	0.1	0.9
DE/rand/1	–	0.9

The results characterized by the mean (the variable ${\rm Mean}$) and the standard deviation (the variable ${\rm Std}.$) are summarized in Table 4 for the set of versions of both the proposed HDE and DE algorithms and the original GWO. In addition, the rank of all algorithms corresponding to each test function is shown in Table 4. The symbols plus sign ‘ $+$’ or minus sign ‘ $-$‘ and the symbol ‘ $=$’ help the readers determine the effectiveness of all proposed HDE compared to their original DE versions. The sign ‘ $+$’ indicates that our proposed algorithm performs better, the sign ‘ $-$’ indicates that it performs worse, and the symbol ‘ $=$’ demonstrates that it performs similarly.

**Table 4:** A comparison of the proposed HDE, enhanced DE algorithms, and GWP for the CEC-2014 test functions.

Optimizer	F1			F2			F3		
	Mean	Std.	*R*/Win	Mean	Std.	*R*/Win	Mean	Std.	*R*/Win
HDE/rand-to-best/2	6.39E+06	4.09E+06	7/+	2.69E+03	4.13E+03	5/−	7.25E+02	2.98E+02	3/+
DE/rand-to-best/2	1.45E+07	6.51E+06	10	5.65E+02	1.09E+03	4	7.17E+03	4.13E+03	11
HDE/current-to-best/1	1.40E+06	7.22E+05	2/+	8.77E+03	1.16E+04	7/+	1.98E+03	2.10E+03	7/+
DE/current-to-best/1	1.20E+07	7.42E+06	9	1.59E+06	2.05E+06	11	3.68E+03	3.36E+03	9
HDE/rand-to-best/1	3.49E+06	2.96E+06	3/+	7.48E+03	6.23E+03	6/+	3.65E+03	2.77E+03	8/+
DE/rand-to-best/1	4.81E+07	2.75E+07	13	3.53E+07	3.21E+07	13	1.22E+04	6.41E+03	13
HDE/current-to-rand/1	2.24E+07	8.83E+06	11/+	1.99E+08	1.17E+08	14/+	1.94E+03	2.73E+03	6/+
DE/current-to-rand/1	2.58E+08	8.77E+07	17	7.18E+09	2.62E+09	16	2.56E+04	9.76E+03	14
HDE/best/2	4.17E+06	1.83E+06	5/+	1.89E+02	2.49E+02	2/−	1.94E+02	1.08E+02	1/+
DE/best/2	1.06E+07	1.55E+07	8	1.39E+02	2.03E+02	1	5.13E+03	2.85E+03	10
HDE/best/1	1.09E+06	1.07E+06	1/+	1.96E+02	3.20E+02	3/+	1.93E+03	1.47E+03	5/+
DE/best/1	1.11E+08	4.23E+07	15	1.28E+10	6.28E+09	17	9.32E+04	5.80E+04	17
HDE/rand/2	5.38E+06	2.83E+06	6/+	7.06E+04	3.20E+04	10/+	1.36E+03	1.17E+03	4/+
DE/rand/2	2.16E+08	3.71E+07	16	4.58E+06	6.25E+06	12	4.26E+04	1.45E+04	16
HDE/rand/1	3.95E+06	3.50E+06	4/+	1.30E+04	1.40E+04	9/−	6.71E+02	4.36E+02	2/+
DE/rand/1	4.08E+07	1.59E+07	12	1.04E+04	1.31E+04	8	9.46E+03	2.03E+03	12
GWO	9.35E+07	7.56E+07	14	2.70E+09	2.46E+09	15	2.81E+04	1.12E+04	15

Optimizer	F4			F5			F6		
	Mean	Std.	*R*/Win	Mean	Std.	*R*/Win	Mean	Std.	*R*/Win
HDE/rand-to-best/2	1.49E+01	3.01E+01	4/–	20.939	9.78E–02	8/+	4.23E+00	2.66E+00	5/–
DE/rand-to-best/2	1.30E+01	2.82E+01	3	20.975	5.00E–02	14	3.59E+00	2.78E+00	4
HDE/current-to-best/1	9.27E+01	3.02E+01	7/+	20.928	4.31E–02	7/+	6.68E+00	1.71E+00	6/+
DE/current-to-best/1	1.45E+02	4.10E+01	12	20.951	3.19E–02	9	7.30E+00	1.35E+00	8
HDE/rand-to-best/1	1.24E+02	2.79E+01	10/+	20.979	4.11E–02	15/–	9.95E+00	3.22E+00	11/–
DE/rand-to-best/1	1.61E+02	5.38E+01	14	20.955	7.48E–02	12	7.09E+00	7.77E–01	7
HDE/current-to-rand/1	1.60E+02	4.11E+02	13/+	20.954	5.69E–02	11/+	8.52E+00	1.71E+00	9/+
DE/current-to-rand/1	7.67E+02	1.88E+02	16	20.999	9.55E–03	16	1.27E+01	8.91E–01	13
HDE/best/2	2.89E+01	3.20E+01	5/+	20.925	5.59E–02	5/=	8.73E+00	2.30E+00	10/+
DE/best/2	3.98E+01	3.58E+01	6	20.925	5.27E–02	5	1.11E+01	2.84E+00	12
HDE/best/1	1.06E+02	3.09E+01	8/+	20.975	7.91E–03	13/–	1.38E+01	1.28E+00	14/+
DE/best/1	1.27E+03	6.92E+02	17	20.9122	4.78E–02	2	2.04E+01	3.53E+00	16
HDE/rand/2	1.15E+02	1.77E+01	9/+	20.917	7.52E–02	4/+	2.29E+00	1.58E+00	1/+
DE/rand/2	1.33E+02	6.50E+00	11	20.9272	1.75E–02	6	3.64E+01	1.59E+00	17
HDE/rand/1	9.65E–01	1.30E–01	2/–	20.9535	4.22E–02	10/–	2.62E+00	1.59E+00	2/+
DE/rand/1	8.02E–01	8.88E–02	1	20.917	4.18E–02	3	3.09E+00	1.26E+00	3
GWO	2.53E+02	4.61E+0	15	20.8864	7.68E–02	1	1.55E+01	3.81E+00	15

Optimizer	F7			F8			F9		
	Mean	Std.	*R*/Win	Mean	Std.	*R*/Win	Mean	Std.	*R*/Win
HDE/rand-to-best/2	6.90E−03	9.61E−02	4/+	1.99E+01	5.54E+00	2/+	6.24E+01	6.05E+01	4/+
DE/rand-to-best/2	7.39E−03	1.18E−02	5	1.70E+02	8.61E+00	16	2.04E+02	8.08E+00	15
HDE/current-to-best/1	7.88E−03	1.28E−02	6/+	3.20E+01	8.00E+00	3/+	4.50E+01	6.15E+00	1/+
DE/current-to-best/1	5.67E−01	5.62E−01	11	3.66E+01	1.60E+01	6	1.02E+02	4.06E+01	8
HDE/rand-to-best/1	1.92E−02	3.10E−02	8/+	3.46E+01	9.32E+00	5/+	1.09E+02	7.50E+01	9/−
DE/rand-to-best/1	1.56E+00	1.17E+00	13	4.17E+01	1.28E+01	7	4.80E+01	1.07E+01	2
HDE/current-to-rand/1	2.02E+00	5.66E−01	14/+	1.37E+01	7.19E+00	1/+	7.96E+01	6.53E+01	6/−
DE/current-to-rand/1	1.05E+02	2.81E+01	16	3.41E+01	9.46E+00	4	5.93E+01	2.17E+01	3
HDE/best/2	5.92E−03	5.40E−03	3/+	7.57E+01	6.91E+01	9/+	1.39E+02	9.33E+01	10/+
DE/best/2	8.87E−03	1.01E−02	7	1.60E+02	1.26E+01	15	2.33E+02	2.13E+01	16
HDE/best/1	2.99E−02	3.33E−02	9/+	4.68E+01	1.19E+01	8/+	6.95E+01	4.54E+00	5/+
DE/best/1	1.33E+02	7.20E+01	17	1.08E+02	3.04E+01	11	1.76E+02	4.24E+01	13
HDE/rand/2	4.27E−01	1.88E−01	10/+	1.39E+02	2.60E+01	13/+	1.57E+02	6.20E+01	11/+
DE/rand/2	6.56E−01	2.53E−01	12	2.05E+02	1.15E+01	17	2.43E+02	1.56E+01	17
HDE/rand/1	4.55E−14	6.22E−14	1/+	1.34E+02	5.83E+00	12/+	1.71E+02	9.31E+00	12/+
DE/rand/1	6.82E−14	6.23E−14	2	1.43E+02	1.76E+01	14	1.98E+02	4.71E+00	14
GWO	2.11E+01	2.03E+01	15	8.15E+01	1.11E+01	10	9.00E+01	2.40E+01	7

Optimizer	F10			F11			F12		
	Mean	Std.	*R*/Win	Mean	Std.	*R*/Win	Mean	Std.	*R*/Win
HDE/rand-to-best/2	1.31E+03	1E71+03	6/+	4.81E+03	2.47E+03	7/+	2.03E+00	4.57E−01	2/+
DE/rand-to-best/2	4.31E+03	3.69E+03	15	6.89E+03	4.34E+02	15	2.45E+00	4.01E−01	8
HDE/current-to-best/1	9.43E+02	1.19E+03	5/+	1.87E+03	5.70E+02	2/+	2.46E+00	2.53E−01	9/+
DE/current-to-best/1	1.63E+03	1.66E+03	9	6.38E+03	4.15E+02	11	2.63E+00	1.67E−01	16
HDE/rand-to-best/1	8.52E+02	5.68E+02	4/−	4.84E+03	2.44E+03	8/−	2.42E+00	2.49E−01	7/+
DE/rand-to-best/1	8.17E+02	1.68E+02	3	4.14E+03	2.08E+03	6	2.54E+00	2.13E−01	13
HDE/current-to-rand/1	4.03E+02	2.62E+02	2/−	1.57E+03	2.73E+02	1/+	2.50E+00	2.15E−01	12/+
DE/current-to-rand/1	3.56E+02	2.06E+02	1	5.83E+03	3.26E+02	10	2.55E+00	1.65E−01	14
HDE/best/2	1.43E+03	5.83E+02	8/+	5.82E+03	2.14E+03	9/+	2.62E+00	2.13E−01	15/−
DE/best/2	1.94E+03	2.71E+02	10	6.54E+03	6.79E+02	12	2.38E+00	1.21E−01	5
HDE/best/1	1.33E+03	5.64E+02	7/+	2.50E+03	6.31E+02	3/+	2.30E+00	5.28E−01	4/−
DE/best/1	3.07E+03	4.54E+02	12	3.15E+03	6.56E+02	5	2.13E+00	1.06E+00	3
HDE/rand/2	5.28E+03	1.52E+02	17/−	6.73E+03	1.85E+02	13/+	2.64E+00	2.45E−01	17/−
DE/rand/2	5.21E+03	2.47E+02	16	7.02E+03	3.71E+02	17	2.48E+00	2.98E−01	10
HDE/rand/1	3.89E+03	2.40E+02	14/−	6.85E+03	1.12E+02	14/+	2.41E+00	5.37E−01	6/+
DE/rand/1	3.57E+03	5.22E+02	13	6.97E+03	2.70E+02	16	2.49E+00	1.73E−01	11
GWO	2.26E+03	4.09E+02	11	2.83E+03	6.87E+02	4	5.95E−01	9.62E−01	1

Optimizer	F13			F14			F15		
	Mean	Std.	*R*/Win	Mean	Std.	*R*/Win	Mean	Std.	*R*/Win
HDE/rand-to-best/2	2.68E−01	2.48E−02	3/+	4.01E−01	1.94E−01	6/−	1.32E+01	4.95E+00	6/+
DE/rand-to-best/2	3.96E−01	5.78E−02	11	3.39E−01	2.37E−01	3	1.72E+01	1.57E+00	12
HDE/current-to-best/1	2.72E−01	1.15E−01	4/+	3.63E−01	1.57E−01	4/−	6.71E+00	5.01E+00	2/+
DE/current-to-best/1	3.20E−01	9.20E−02	7	3.34E−01	1.71E−01	2	1.18E+01	1.45E+00	4
HDE/rand-to-best/1	2.61E−01	3.69E−02	2/+	5.34E−01	1.78E−01	10/−	1.22E+01	4.81E+00	5/−
DE/rand-to-best/1	3.63E−01	9.39E−02	8	3.82E−01	1.90E−01	5	9.42E+00	7.09E+00	3
HDE/current-to-rand/1	2.52E−01	5.58E−02	1/+	3.19E−01	2.20E−02	1/+	1.45E+01	4.62E+00	7/+
DE/current-to-rand/1	2.45E+00	1.90E−01	16	2.70E+01	5.62E+00	16	1.16E+03	1.44E+03	16
HDE/best/2	4.70E−01	5.21E−02	12/+	4.09E−01	2.10E−01	7/+	1.47E+01	4.03E+00	8/+
DE/best/2	5.41E−01	6.78E−02	13	8.33E−01	3.45E−01	14	1.77E+01	7.87E−01	13
HDE/best/1	2.96E−01	3.96E−02	6/+	5.56E−01	3.48E−01	11/+	6.10E+00	2.37E+00	1/+
DE/best/1	3.03E+00	9.44E−01	17	4.99E+01	3.52E+01	17	7.41E+04	7.77E+04	17
HDE/rand/2	3.84E−01	6.09E−02	9/+	5.62E−01	2.52E−01	12/+	1.58E+01	6.77E−01	10/+
DE/rand/2	6.77E−01	4.57E−02	14	8.08E−01	2.99E−01	13	2.30E+01	1.29E+00	14
HDE/rand/1	2.87E−01	8.49E−02	5/+	4.34E−01	1.77E−01	8/+	1.54E+01	9.04E−01	9/+
DE/rand/1	3.96E−01	6.90E−02	10	4.34E−01	2.34E−01	9	1.68E+01	6.62E−01	11
GWO	8.19E−01	9.68E−01	15	1.15E+00	1.74E+00	15	4.64E+02	9.12E+02	15

Optimizer	F16			F17			F18		
	Mean	Std.	*R*/Win	Mean	Std.	*R*/Win	Mean	Std.	*R*/Win
HDE/rand-to-best/2	1.07E+01	5.87E−01	3/+	5.42E+05	3.40E+05	7/+	5.70E+03	6.64E+03	13/−
DE/rand-to-best/2	1.25E+01	3.88E−01	14	5.83E+05	3.87E+05	8	3.26E+03	4.43E+03	6
HDE/current-to-best/1	1.09E+01	4.03E−01	5/+	3.12E+05	1.84E+05	4/+	3.92E+03	2.77E+03	8/+
DE/current-to-best/1	1.14E+01	4.23E−01	9	7.93E+05	4.50E+05	12	4.45E+03	5.35E+03	10
HDE/rand-to-best/1	1.12E+01	2.22E−01	8/−	4.98E+05	2.93E+05	6/+	4.20E+03	4.13E+03	9/−
DE/rand-to-best/1	1.10E+01	5.79E−01	6	1.03E+06	2.65E+05	13	3.42E+03	2.62E+03	7
HDE/current-to-rand/1	1.05E+01	2.15E−01	1/+	7.78E+05	5.03E+05	11/+	2.95E+03	3.62E+03	3/+
DE/current-to-rand/1	1.10E+01	3.34E−01	7	4.81E+06	2.90E+06	16	1.85E+07	2.13E+07	17
HDE/best/2	1.17E+01	3.58E−01	12/+	1.61E+05	5.59E+04	1/+	5.04E+03	3.58E+03	11/+
DE/best/2	1.23E+01	2.65E−01	13	2.02E+05	2.13E+05	2	7.98E+03	6.35E+03	14
HDE/best/1	1.07E+01	4.04E−01	2/+	2.85E+05	3.34E+05	3/+	5.67E+03	5.56E+03	12/+
DE/best/1	1.15E+01	4.05E−01	10	3.65E+06	2.86E+06	15	1.17E+06	2.58E+06	15
HDE/rand/2	1.28E+01	4.03E−01	15/+	6.10E+05	2.41E+05	9/+	3.12E+03	3.70E+03	4/+
DE/rand/2	1.31E+01	1.00E−01	17	6.00E+06	1.78E+06	17	3.14E+03	2.98E+03	5
HDE/rand/1	1.15E+01	4.82E−01	11/+	6.10E+05	2.73E+05	10/+	6.16E+02	5.53E+02	1/+
DE/rand/1	1.28E+01	1.02E−01	16	1.77E+06	9.15E+05	14	9.29E+02	3.87E+02	2
GWO	1.08E+01	8.11E−01	4	4.91E+05	3.94E+05	5	2.06E+06	4.57E+06	16

Optimizer	F19			F20			F21		
	Mean	Std.	*R*/Win	Mean	Std.	*R*/Win	Mean	Std.	*R*/Win
HDE/rand-to-best/2	8.11E+00	2.11E+00	4/−	3.06E+02	1.44E+02	3/+	1.10E+05	8.21E+04	4/+
DE/rand-to-best/2	7.83E+00	1.80E+00	3	1.74E+03	8.40E+02	10	1.39E+05	7.02E+04	5
HDE/current-to-best/1	8.73E+00	1.43E+00	7/+	2.04E+02	9.01E+01	1/+	1.97E+05	1.38E+05	7/+
DE/current-to-best/1	1.48E+01	7.62E+00	13	2.91E+02	1.25E+02	2	2.60E+05	2.40E+05	10
HDE/rand-to-best/1	7.05E+00	9.28E−01	1/+	1.65E+03	2.79E+03	9/+	2.43E+05	2.06E+05	9/+
DE/rand-to-best/1	1.42E+01	5.05E+00	12	8.50E+03	6.63E+03	13	6.81E+05	6.05E+05	14
HDE/current-to-rand/1	2.74E+01	2.43E+01	15/+	3.64E+02	1.31E+02	4/+	3.56E+05	2.49E+05	12/+
DE/current-to-rand/1	1.40E+02	2.55E+01	17	1.51E+04	3.57E+03	15	1.05E+06	7.26E+05	16
HDE/best/2	8.47E+00	1.78E+00	6/+	4.11E+02	7.55E+01	5/+	4.31E+04	2.01E+04	1/+
DE/best/2	9.97E+00	2.67E+00	9	2.34E+03	1.78E+03	11	9.49E+04	8.44E+04	3
HDE/best/1	1.11E+01	2.44E+00	11/+	5.74E+02	5.77E+02	6/+	6.24E+04	3.52E+04	2/+
DE/best/1	7.42E+01	4.08E+01	16	2.71E+04	3.00E+04	17	7.40E+05	7.73E+05	15
HDE/rand/2	1.02E+01	2.89E+00	10/−	6.15E+02	2.26E+02	8/+	2.30E+05	1.44E+05	8/+
DE/rand/2	9.73E+00	2.28E+00	8	1.31E+04	3.52E+03	14	2.07E+06	1.60E+06	17
HDE/rand/1	8.28E+00	4.02E−01	5/−	6.04E+02	4.94E+02	7/+	1.72E+05	1.14E+05	6/+
DE/rand/1	7.47E+00	1.05E+00	2	3.17E+03	1.15E+03	12	3.09E+05	3.05E+05	11
GWO	2.64E+01	1.42E+01	14	2.01E+04	9.66E+03	16	5.66E+05	5.63E+05	13

Optimizer	F22			F23			F24		
	Mean	Std.	*R*/Win	Mean	Std.	*R*/Win	Mean	Std.	*R*/Win
HDE/rand-to-best/2	3.75E+02	2.48E+02	14/−	3.152E+02	2.050E−13	1/=	2.000E+02	1.200E−03	5/+
DE/rand-to-best/2	2.69E+02	1.56E+02	7	3.152E+02	0.000E+00	1	2.246E+02	2.851E+00	10
HDE/current-to-best/1	2.10E+02	1.66E+02	3/+	3.152E+02	5.419E−12	1/+	2.000E+02	1.470E−03	3/+
DE/current-to-best/1	3.01E+02	1.32E+02	9	3.191E+02	4.709E+00	5	2.399E+02	8.193E+00	14
HDE/rand-to-best/1	2.28E+02	1.50E+02	5/+	3.152E+02	1.364E−03	2/+	2.000E+02	1.532E−03	4/+
DE/rand-to-best/1	3.07E+02	1.30E+02	10	3.198E+02	2.760E+00	6	2.416E+02	9.363E+00	16
HDE/current-to-rand/1	2.14E+02	6.55E+01	4/+	3.200E+02	2.387E+00	7/+	2.313E+02	7.879E+00	12/+
DE/current-to-rand/1	3.71E+02	5.85E+01	13	3.681E+02	1.261E+01	9	2.411E+02	3.807E+00	15
HDE/best/2	3.33E+02	3.07E+02	11/−	3.152E+02	6.430E−13	1/=	2.132E+02	1.203E+01	7/+
DE/best/2	2.99E+02	1.13E+02	8	3.154E+02	7.610E−13	1	2.345E+02	9.325E+00	13
HDE/best/1	3.34E+02	1.99E+02	12/+	3.152E+02	6.225E−11	1/+	2.000E+02	7.301E−04	1/+
DE/best/1	6.16E+02	1.54E+02	17	3.992E+02	7.658E+01	10	3.042E+02	2.660E+01	17
HDE/rand/2	4.17E+02	7.54E+01	15/+	3.152E+02	2.732E−04	3/+	2.000E+02	1.495E−02	6/+
DE/rand/2	4.54E+02	7.64E+01	16	3.153E+02	2.609E−02	4	2.256E+02	6.207E+00	11
HDE/rand/1	1.51E+02	1.00+02	1/+	3.152E+02	4.069E−13	1/=	2.173E+02	9.677E+00	8/+
DE/rand/1	2.02E+02	9.44E+01	2	3.152E+02	7.610E−13	1	2.242E+02	2.441E+00	9
GWO	2.31E+02	1.33E+02	6	3.322E+02	6.840E+00	8	2.000E+02	1.103E−03	2

Optimizer	F25			F26			F27		
	Mean	Std.	*R*/Win	Mean	Std.	*R*/Win	Mean	Std.	*R*/Win
HDE/rand-to-best/2	2.04E+02	6.92E−01	3/+	1.408E+02	9.060E+01	12/+	3.67E+02	7.31E+01	4/+
DE/rand-to-best/2	2.06E+02	1.37E+00	4	1.408E+02	9.057E+01	13	4.85E+02	1.35E+02	7
HDE/current-to-best/1	2.06E+02	1.57E+00	8/+	1.002E+02	4.410E−02	2/+	4.91E+02	7.43E+01	8/+
DE/current-to-best/1	2.09E+02	2.25E+00	10	1.405E+02	5.502E+01	11	5.81E+02	1.25E+02	10
HDE/rand-to-best/1	2.06E+02	1.06E+00	7/+	1.801E+02	4.465E+01	15/−	4.61E+02	5.93E+01	6/+
DE/rand-to-best/1	2.12E+02	3.89E+00	14	1.404E+02	5.497E+01	10	5.78E+02	1.19E+02	9
HDE/current-to-rand/1	2.13E+02	2.29E+00	15/−	1.003E+02	5.092E−02	3/+	6.13E+02	5.82E+01	13/−
DE/current-to-rand/1	2.11E+02	8.84E−01	12	1.717E+02	4.140E+01	14	5.94E+02	7.77E+01	11
HDE/best/2	2.03E+02	5.25E−01	1/+	1.003E+02	4.565E−02	6/+	6.00E+02	1.38E+02	12/+
DE/best/2	2.06E+02	2.73E+00	6	1.006E+02	5.557E−02	8	6.91E+02	4.01E+01	14
HDE/best/1	2.07E+02	2.63E+00	9/+	1.201E+02	4.425E+01	9/+	7.09E+02	8.41E+01	15/+
DE/best/1	2.24E+02	6.67E+00	16	1.847E+02	4.566E+01	16	9.76E+02	2.08E+02	17
HDE/rand/2	2.06E+02	1.58E+00	5/+	1.003E+02	8.067E−02	4/+	3.99E+02	5.31E+01	5/+
DE/rand/2	2.40E+02	1.35E+01	17	1.006E+02	7.450E−02	7	7.33E+02	1.90E+02	16
HDE/rand/1	2.03E+02	7.93E−01	2/+	1.002E+02	3.539E−02	1/+	5.18E+01	2.93E+01	1/+
DE/rand/1	2.12E+02	9.69E−01	13	1.003E+02	4.645E−02	5	3.55E+02	4.74E+01	3
GWO	2.11E+02	3.25E+00	11	2.000E+02	3.166E−02	17	6.05E+01	7.15E+01	2

Optimizer	F28			F29			F30		
	Mean	Std.	*R*/Win	Mean	Std.	*R*/Win	Mean	Std.	*R*/Win
HDE/rand-to-best/2	8.55E+02	2.42E+01	6/−	1.79E+06	3.99E+06	12/−	3.30E+03	8.10E+02	2/+
DE/rand-to-best/2	8.22E+02	7.20E+01	2	1.78E+06	3.99E+06	11	2.39E+04	4.63E+02	13
HDE/current-to-best/1	8.83E+02	8.53E+01	8/+	1.67E+03	6.35E+02	2/+	3.50E+03	1.49E+03	4/+
DE/current-to-best/1	1.18E+03	1.31E+02	15	3.68E+06	5.04E+06	14	7.33E+03	2.26E+03	11
HDE/rand-to-best/1	8.99E+02	1.88E+01	9/+	1.90E+03	7.32E+02	3/+	4.18E+03	7.60E+02	10/+
DE/rand-to-best/1	1.36E+03	3.87E+02	16	1.47E+06	3.25E+06	10	2.09E+04	1.59E+04	12
HDE/current-to-rand/1	9.61E+02	1.09E+02	10/+	7.67E+04	1.27E+05	9/+	3.68E+04	3.52E+04	14/+
DE/current-to-rand/1	1.13E+03	1.55E+02	14	8.76E+06	8.74E+06	17	2.45E+05	1.47E+05	16
HDE/best/2	1.03E+03	1.74E+02	11/+	1.47E+03	3.17E+02	1/+	3.54E+03	1.59E+03	6/+
DE/best/2	1.12E+03	1.41E+02	13	4.11E+06	5.71E+06	15	3.69E+03	9.66E+02	8
HDE/best/1	1.07E+03	2.00E+02	12/+	2.39E+03	1.15E+03	6/+	3.62E+03	4.68E+02	7/+
DE/best/1	2.26E+03	5.47E+02	17	3.17E+06	4.36E+06	13	2.56E+05	1.79E+05	13/+
HDE/rand/2	8.27E+02	1.40E+01	3/+	2.37E+03	6.75E+02	5/+	3.27E+03	2.36E+02	1/+
DE/rand/2	8. 60E+02	9.65E+01	7	2.55E+03	8.41E+02	7	3.30E+03	4.01E+02	3
HDE/rand/1	8.32E+02	1.29E+01	4/+	2.84E+03	7.36E+01	8/−	3.89E+03	4.40E+02	9/−
DE/rand/1	8.53E+02	1.80E+01	5	2.28E+03	7.87E+02	4	3.53E+03	1.14E+03	5
GWO	1.98E+02	1.57E+02	1	5.09E+06	4.65E+06	16	5.29E+04	3.39E+04	15

Table 5 shows a global summary of the ranking sums (variable “ ${\rm SRN}$”) of all optimization algorithms from Table 4, then their qualitative ranking according to the value of ${\rm SRN}$, and the mean rank in the Friedman test. Furthermore, in Table 5, we find qualitative rankings by the partial summary of the ranking sums ‘ ${\rm SRN} - {\rm UF}$’, ‘ ${\rm SRN} - {\rm SMF}$’, ‘ ${\rm SRN} - {\rm HF}$’, and ‘ ${\rm SRN} - {\rm CF}$’ for subgroups of functions unimodal function (UF), simple multimodal function (SMF), hybrid function (HF), and composition function (CF), respectively. Table 5 implies that the three methods that globally best optimize the CEC-2014 benchmark functions are “HDE/current-to-best/1”, “HDE/rand-to-best/2” and “HDE/rand/1”.

**Table 5:** Analysis of optimizers based on rankings for CEC-2014 functions.

Method	Rank by ‘ ${\bf SRN} - {\bf UF}$’	Rank by ‘SRN-SMF’	Rank by ‘SRN-HF’	Rank by ‘SRN-CF’	Sum of all ranks (SRN)	Rank by ‘SRN’	Friedman test value
HDE/rand-to-best/2	3.5	1	7	5	165	2	5.63
DE/rand-to-best/2	9	12	4.5	9	260	10	8.85
HDE/current-to-best/1	5	2	1.5	3	143	1	4.87
DE/current-to-best/1	10	8	12	14	287	12	9.83
HDE/rand-to-best/1	6	6	4.5	7	214	5	7.22
DE/rand-to-best/1	13	4	13	15	300	13	10.27
HDE/current-to-rand/1	11	3	10	13	242	7	8.38
DE/current-to-rand/1	16	15	16	16	397	16	13.47
HDE/best/2	1	9	3	5	201	4	6.82
DE/best/2	7	13.5	9	12	285	11	9.83
HDE/best/1	2	5	8	8	216	6	7.15
DE/best/1	17	16	17	17	420	17	14.38
HDE/rand/2	8	13.5	11	1	247	9	8.28
DE/rand/2	14.5	17	15	10.5	374	15	12.7
HDE/rand/1	3.5	7	1.5	2	185	3	6.32
DE/rand/1	12	10	6	5	243	8	8.23
GWO	14.5	11	14	10.5	314	14	10.77

Further concretely, that:

- “HDE/best/2”, “HDE/best/1”, “HDE/rand-to-best/2”, and “HDE/rand/1” best optimize the subgroup UF,
- “HDE/rand-to-best/2”, "HDE/current-to-best/1”, and “HDE/current-to-rand/1” best optimize the subgroup SMF,
- “HDE/current-to-best/1”, “HDE/rand/1”, and “HDE/best/2” best optimize the subgroup HF,
- “HDE/rand/2”, “HDE/rand/1”, and “HDE/current-to-best/1” best optimize the subgroup CF.

The list of previous items shows that “HDE/current-to-best/1” and “HDE/rand/1” were the best optimizers three times placed top three positions in all four subgroups of CEC-2017 (similarly, “HDE/rand-to-best/2” two times placed top three positions).

In addition to the already mentioned great results of “HDE/current-to-best/1”, “HDE/rand-to-best/2”, and “HDE/rand/1” in some subsets of CEC-2017, it should be added that neither they did not do poorly on the other subsets, as they always ranked above the median, what indicates the robustness and dependability of the proposed HDE algorithms.

**Figure 1:**
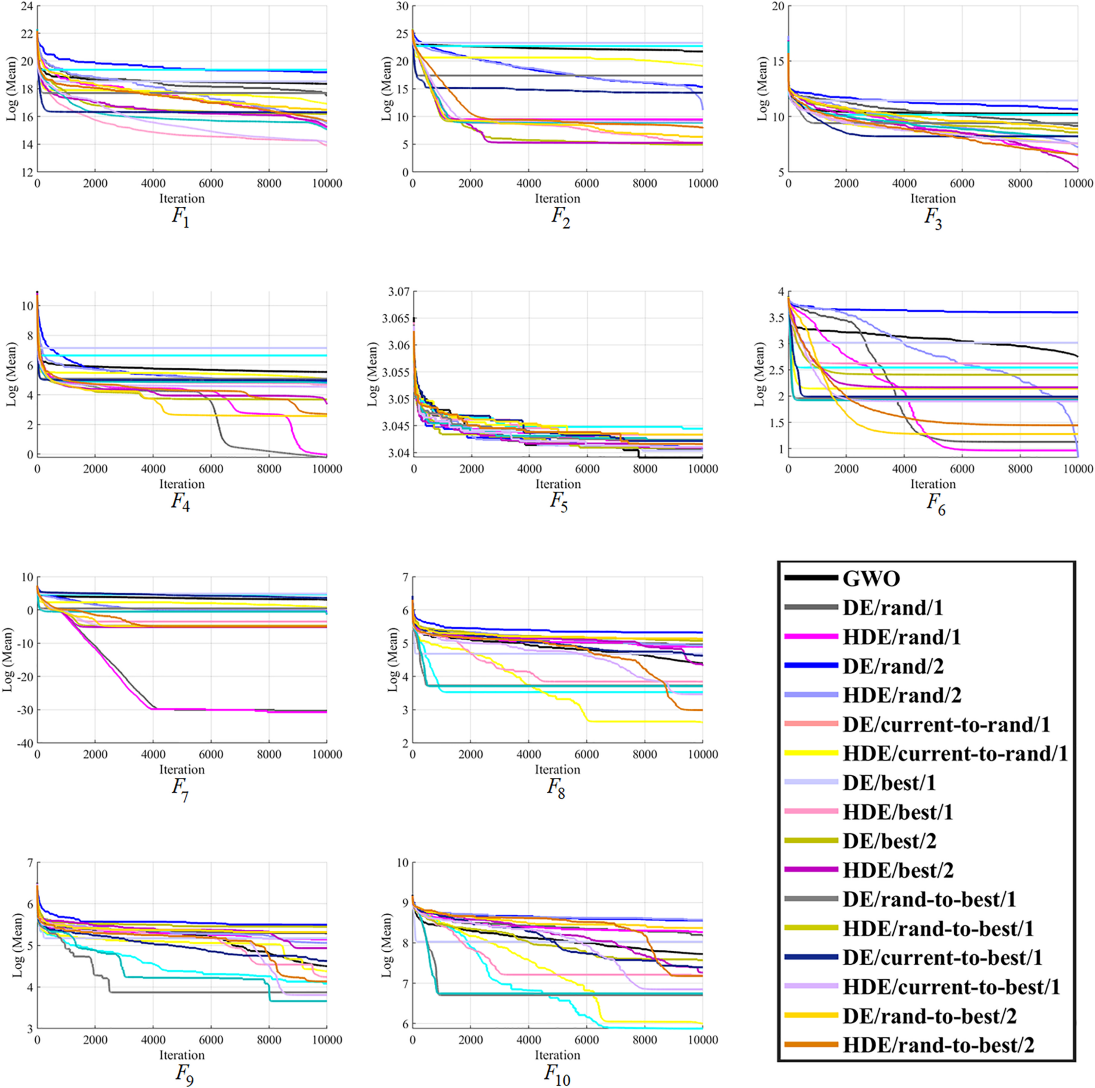
The optimizing process of all the studied optimizers for the benchmark function F1–F10 from the CEC-2014 suite.

**Figure 2:**
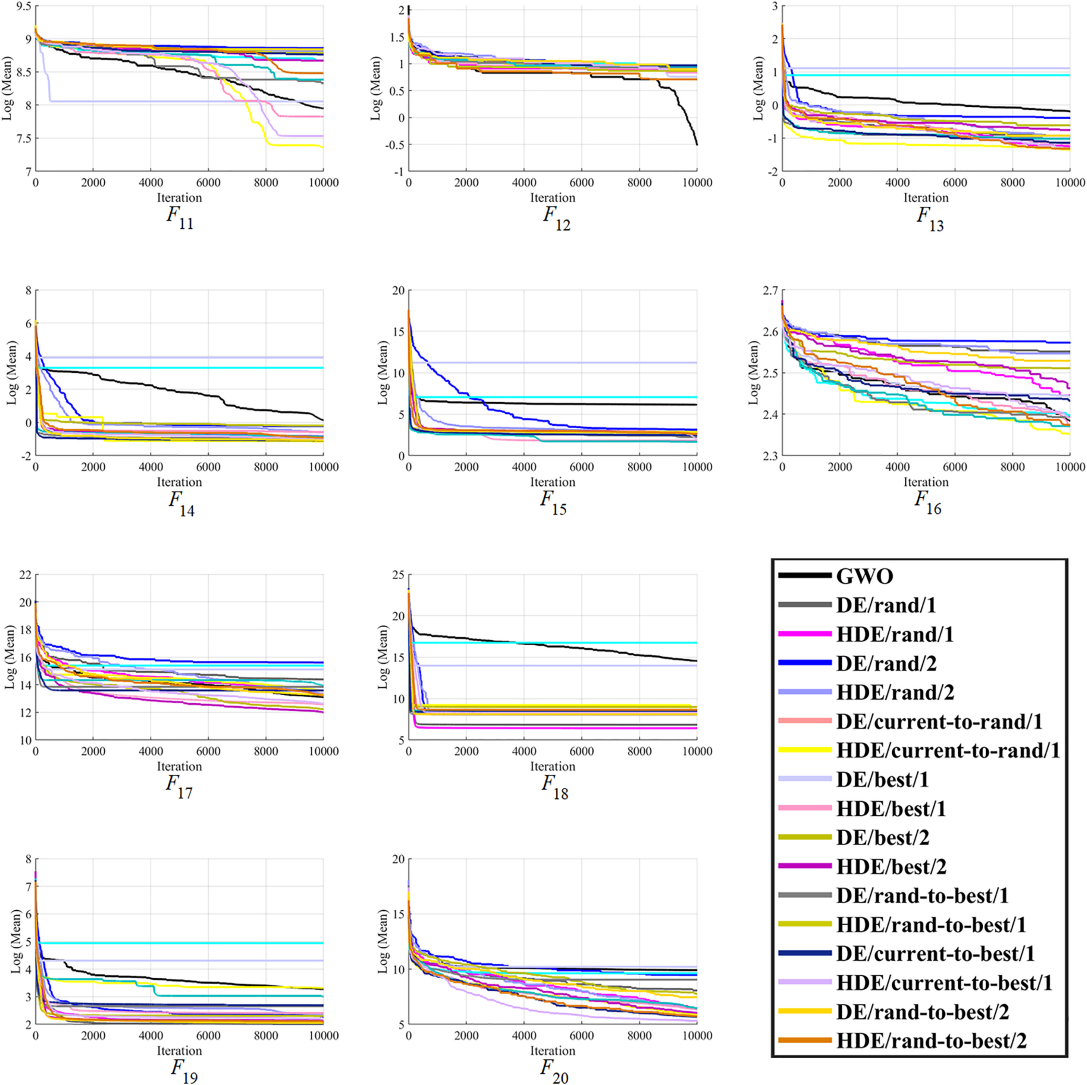
The optimizing process of all the studied optimizers for the benchmark function F11–F20 from the CEC-2014 suite.

**Figure 3:**
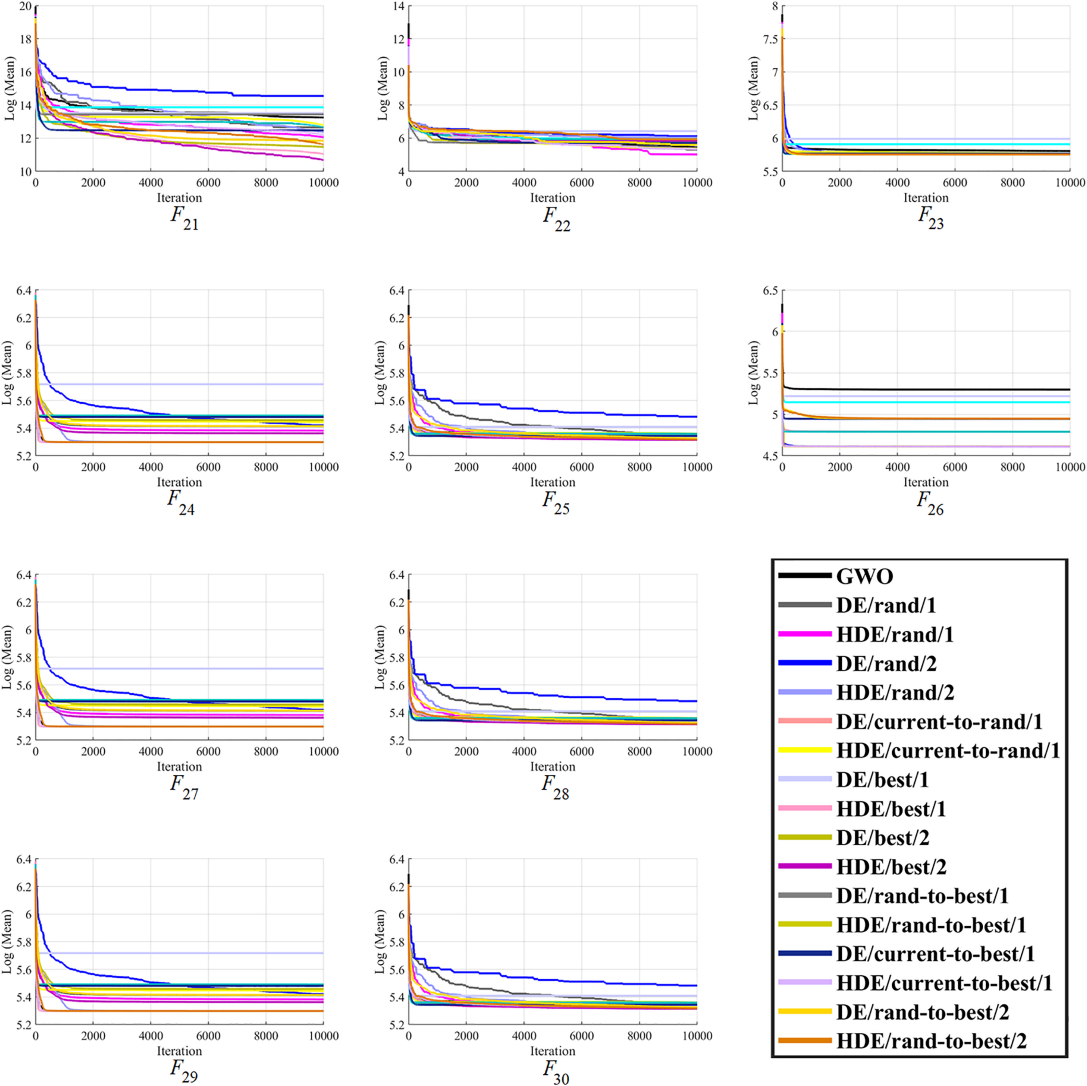
The optimizing process of all the studied optimizers for the benchmark function F21–F30 from the CEC-2014 suite.

Moreover, finally, it can be seen that the proposed HDEs are generally much more successful than their original DE versions, proving their effectiveness and superiority over original DE versions. Hence, it is fair to say that the proposed HDE algorithms produce respectable solutions with the potential for further optimization toward becoming a more reliable algorithm. Figures 1–3 present good convergence properties of the HDEs on 30 test functions from the CEC-2014.

The outcomes of applying various proposed techniques to the CEC-2019 suite benchmark functions are shown in Table 6. The Friedman test demonstrates that the three best algorithms dealing with this set of benchmark functions are from the proposed HDEs, confirming the proposed method's effectiveness. The “HDE/current-to-rand/1” and the “HDE/rand-to-best/1” perform best on the CEC-2019 suite benchmark functions.

**Table 6:** The achieved optimal results using HDEs for the CEC-2019 test suite.

Function	GWO	HDE/rand/1	HDE/rand/2	HDE/current-to-rand/1	HDE/best/1	HDE/best/2	HDE/rand-to-best/1	HDE/current-to-best/1	HDE/rand-to-best/2
F1	5.88E+03 3.04E+04	2.85E+05 3.08E+05	1.81E+05 1.81E+05	8.79E+04 1.53E+05	3.36E+04 1.06E+05	9.60E+04 1.04E+05	1.57E+04 2.91E+04	4.04E+04 7.34E+04	4.16E+04 6.45E+04
F2	1.79E+02 1.48E+02	3.94E+02 1.03E+02	3.87E+02 1.40E+02	3.43E+02 1.36E+02	2.86E+02 1.38E+02	4.69E+02 1.23E+02	3.05E+02 1.49E+02	3.00E+02 1.36E+02	2.83E+02 1.43E+02
F3	1.85E+00 1.34E+00	4.58E+00 1.56E+00	4.79E+00 1.57E+00	1.77E+00 1.30E+00	1.58E+00 1.17E+00	3.92E+00 2.98E+00	1.63E+00 1.08E+00	1.59E+00 1.32E+00	1.90E+00 1.48E+00
F4	1.40E+01 7.59E+00	2.15E+01 5.37E+00	2.71E+01 4.22E+00	6.30E+00 5.16E+00	9.85E+00 6.68E+00	1.76E+01 8.42E+00	8.02E+00 3.27E+00	9.18E+00 6.22E+00	1.35E+01 8.56E+00
F5	1.55E+00 5.77E−01	1.40E+00 1.04E−01	1.53E+00 8.05E−02	1.08E+00 1.05E−01	1.10E+00 6.38E−02	1.37E+00 1.88E−01	1.08E+00 1.08E−01	1.09E+00 8.33E−02	1.45E+00 1.33E−01
F6	2.12E+00 1.19E+00	1.28E+00 2.34E−01	1.96E+00 9.52E−01	1.47E+00 5.20E−01	2.27E+00 9.89E−01	1.80E+00 8.65E−01	1.66E+00 8.19E−01	1.32E+00 6.73E−01	1.42E+00 6.65E−01
F7	6.82E+02 2.56E+02	4.95E+02 3.32E+02	8.19E+02 3.29E+02	3.14E+02 2.25E+02	4.10E+02 2.21E+02	4.92E+02 3.24E+02	3.14E+02 1.68E+02	3.54E+02 2.37E+02	3.75E+02 3.28E+02
F8	3.50E+00 5.45E−01	2.84E+00 3.55E−01	3.27E+00 4.88E−01	2.82E+00 5.49E−01	3.09E+00 4.58E−01	3.44E+00 3.08E−01	3.11E+00 5.57E−01	3.11E+00 5.09E−01	2.88E+00 4.96E−01
F9	1.13E+00 5.35E−02	1.14E+00 3.71E−02	1.17E+00 3.18E−02	1.11E+00 3.46E−02	1.12E+00 4.55E−02	1.16E+00 4.14E−02	1.12E+00 3.62E−02	1.12E+00 3.57E−02	1.14E+00 3.02E−02
F10	2.07E+01 2.33E+00	2.14E+01 7.19E−02	2.14E+01 5.73E−02	1.85E+01 6.99E+00	2.07E+01 3.72E+00	2.13E+01 9.28E−02	1.93E+01 6.21E+00	2.07E+01 3.72E+00	2.00E+01 5.17E+00
Friedman test values	5.7	6.4	8.15	2.7	4.1	7	3.05	3.45	4.45

Also, to compare the proposed HDEs with other evolutionary algorithms, the results of “HDE/current-to-best/1” on the CEC-2014 suite with $D = 30$ are compared to some modified versions of evolutionary algorithms in Table 7, such as m-SCA (Gupta & Deep, 2019a), MG-SCA (Gupta, Deep & Engelbrecht, 2020), CTLBO (Yang, Li & Guo, 2014), LJA (Iacca, dos Santos Junior & de Melo, 2021) and some modified version of GWO, *e.g*., Cauchy-GWO (Gupta & Deep, 2018), RW-GWO (Gupta & Deep, 2019b), and ERGWO (Long et al., 2020). This comparison confirms that the proposed HDE algorithms can be considered an effective evolutionary method dealing with a vast range of optimization problems.

**Table 7:** Comparison between best HDE with some algorithms on the CEC-2014 test suite.

Function		MG-SCA	CTLBO	LJA	m-SCA	RW-GWO	Cauchy- GWO	ERGWO	HDE/current -to-best/1
F1	Unimodal	2.92E+07	2.47E+08	6.31E+07	2.12E+08	8.02E+06	1.70E+07	1.34E +07	1.40E+06
		2.07E+07	3.06E+07	1.87E+07	5.56E+07	3.31E+06	9.07E+06	1.82E +06	7.22E+05
F2		2.26E+09	4.23E+09	4.77E+09	1.57E+10	2.23E+05	3.57E+08	3.41E +06	8.77E+03
		1.69E+09	9.47E+08	6.03E+08	2.48E+09	5.51E+05	7.21E+08	1.18E +06	1.16E+04
F3		1.77E+04	4.31E+04	6.91E+04	3.97E+04	3.16E+02	6.70E+03	1.24E +04	1.98E+03
		6.63E+03	5.32E+03	1.07E+04	7.56E+03	4.34E+02	3.76E+03	2.16E +03	2.10E+03
F4	Simple Multimodal	2.76E+02	5.02E+02	4.08E+02	9.86E+02	3.41E+01	1.32E+02	1.41E +02	9.27E+01
		6.55E+01	5.45E+01	5.38E+01	3.02E+02	1.80E+01	3.52E+01	1.25E +01	3.02E+01
F5		2.04E+01	2.09E+01	2.09E+01	2.09E+01	2.05E+01	2.06E+01	2.05E +01	2.09E+01
		1.44E−01	5.01E−02	4.97E−02	3.78E−02	7.46E−02	3.42E−01	1.71E −02	4.31E−02
F6		1.94E+01	1.73E+01	3.39E+01	3.35E+01	9.84E+00	1.70E+01	1.05E +01	6.68E+00
		2.89E+00	1.86E+00	1.29E+00	2.58E+00	3.49E+00	3.06E+00	2.50E +00	1.71E+00
F7		1.99E+01	4.18E+01	1.58E+01	1.15E+02	2.53E−01	2.22E+00	1.40E +00	7.88E−03
		1.18E+01	5.55E+00	2.80E+00	1.98E+01	1.43E−01	1.87E+00	2.16E−01	1.28E−02
F8		1.07E+02	1.02E+02	2.24E+02	2.36E+02	4.38E+01	7.45E+01	7.37E +01	3.20E+01
		2.14E+01	8.77E+00	9.93E+00	1.48E+01	8.48E+00	1.57E+01	3.07E +01	8.00E+00
F9		1.39E+02	1.85E+02	2.61E+02	2.75E+02	6.33E+01	9.29E+01	8.83E +01	4.50E+01
		2.56E+01	2.92E+01	1.47E+01	1.61E+01	1.30E+01	1.88E+01	2.99E +01	6.15E+00
F10		2.82E+03	2.95E+03	5.68E+03	5.99E+03	9.61E+02	2.59E+03	1.80E +03	9.43E+02
		6.83E+02	5.34E+02	3.95E+02	4.53E+02	2.72E+02	6.14E+02	8.51E +02	1.19E+02
F11		3.30E+03	5.03E+03	6.88E+03	7.00E+03	2.68E+03	3.81E+03	2.51E +03	1.87E+03
		6.26E+02	8.12E+02	3.12E+02	3.42E+02	3.68E+02	5.79E+02	6.88E +02	5.70E+02
F12		6.33E−01	2.08E+00	2.49E+00	2.44E+00	5.45E−01	5.02E−01	1.14E +00	2.46E+00
		3.36E−01	6.74E−01	2.73E−01	3.52E−01	1.66E−01	5.02E−01	8.94E−02	2.53E−01
F13		5.51E−01	9.62E−01	1.08E+00	2.94E+00	2.80E−01	3.56E−01	3.80E−01	2.72E−01
		8.94E−02	6.51E−01	1.19E−01	3.72E−01	6.30E−02	9.08E−02	8.27E−02	1.15E−01
F14		2.34E+00	1.08E+01	4.33E+00	4.42E+01	4.23E−01	4.98E−01	6.40E−01	3.63E−01
		3.31E+00	4.59E+00	1.70E+00	7.81E+00	2.15E−01	2.44E−01	1.95E−01	1.57E−01
F15		8.72E+01	5.26E+01	5.05E+01	1.92E+03	8.81E+00	2.37E+01	2.07E +01	6.71E+00
		1.01E+02	2.29E+01	9.36E+00	1.43E+03	1.51E+00	1.13E+01	2.25E +00	5.01E+00
F16		1.16E+01	1.26E+01	1.28E+01	1.28E+01	1.03E+01	1.09E+01	1.08E +01	1.09E+01
		6.91E−01	2.33E−01	1.78E−01	2.24E−01	6.11E−01	6.31E−01	6.30E−01	4.03E−01
F17	Hybrid	9.56E+05	8.37E+06	2.63E+06	5.37E+06	5.71E+05	3.46E+05	6.76E +05	3.12E+05
		7.62E+05	3.00E+06	9.76E+05	2.76E+06	4.10E+05	2.56E+05	4.14E +05	1.84E+05
F18		1.48E+05	5.51E+02	1.26E+07	1.43E+08	6.52E+03	1.32E+03	2.06E +04	3.92E+03
		9.00E+05	4.71E+02	1.06E+07	8.38E+07	4.62E+03	1.78E+03	1.68E +04	2.77E+03
F19		2.28E+01	7.63E+01	3.78E+01	9.42E+01	1.14E+01	1.80E+01	1.19E +00	8.73E+00
		1.43E+01	9.20E+00	3.46E+01	2.63E+01	2.03E+00	1.04E+01	1.06E +00	1.43E+00
F20		4.24E+03	2.26E+04	9.92E+03	9.51E+03	6.27E+02	9.26E+02	3.59E +03	2.04E+02
		3.82E+03	5.14E+03	3.69E+03	3.62E+03	1.12E+03	1.59E+03	3.21E +03	9.01E+01
F21		2.35E+05	1.31E+06	6.94E+05	1.48E+06	2.58E+05	2.14E+05	1.80E +05	1.97E+05
		2.39E+05	6.39E+05	2.03E+05	6.95E+05	1.76E+05	1.66E+05	4.59E +04	1.38E+05
F22		3.39E+02	5.98E+02	5.47E+02	7.54E+02	2.08E+02	2.39E+02	2.88E +02	2.10E+02
		1.78E+02	1.75E+02	1.05E+02	1.46E+02	1.29E+02	1.19E+02	8.15E +01	1.66E+02
F23	Composition	3.29E+02	3.59E+02	3.43E+02	3.66E+02	3.15E+02	3.18E+02	3.14E +02	3.15E+02
		4.03E+00	3.83E+00	3.41E+00	1.16E+01	2.77E−01	2.38E+00	2.43E−01	5.42E−12
F24		2.00E+02	2.00E+02	2.57E+02	2.00E+02	2.00E+02	2.00E+02	2.00E +02	2.00E+02
		1.56E−03	1.26E+00	4.04E+00	6.94E−02	3.04E−03	2.36E−03	0.00E +00	1.47E−03
F25		2.11E+02	2.00E+02	2.16E+02	2.26E+02	2.04E+02	2.09E+02	2.00E +02	2.06E+02
		2.82E+00	1.33E+00	2.58E+00	8.89E+00	1.18E+00	4.27E+00	0.00E +00	1.57E+00
F26		1.01E+02	1.08E+02	1.01E+02	1.02E+02	1.00E+02	1.00E+02	1.00E +02	1.00E+02
		1.53E−01	2.71E+01	1.02E−01	5.30E−01	7.36E−02	8.20E−02	4.48E−02	4.41E−02
F27		8.19E+02	6.19E+02	9.86E+02	8.28E+02	4.09E+02	4.16E+02	5.71E +02	4.91E+02
		9.17E+01	1.04E+02	2.48E+02	3.39E+02	6.09E+00	9.82E+00	1.03E +02	7.43E+01
F28		9.68E+02	1.79E+03	1.13E+03	1.98E+03	4.34E+02	6.95E+02	9.56E +02	8.83E+02
		1.06E+02	7.89E+02	6.63E+01	2.96E+02	8.45E+00	1.79E+02	7.12E +01	8.53E+01
F29		1.19E+06	3.75E+06	9.82E+05	1.04E+07	2.14E+02	3.33E+02	1.41E +04	1.67E+03
		3.25E+06	7.35E+06	2.07E+06	5.39E+06	2.37E+00	9.43E+01	8.29E +03	6.35E+02
F30		1.92E+04	3.40E+05	1.09E+04	2.38E+05	6.69E+02	1.40E+03	3.16E +04	3.50E+03
		8.25E+03	6.19E+04	4.24E+03	1.01E+05	2.14E+02	4.92E+02	1.25E +04	1.49E+03

The Wilcoxon signed-rank test is designed to test the equality of the medians of two data sets (*e.g*., the results of two algorithms), where this equality would imply that they are interchangeable. The Wilcoxon signed-rank test is based on the magnitude of the score. First, the signed differences are computed through all benchmark functions for a pair of algorithms (in our case, the number of these benchmark functions equals 30). Because the range of benchmark functions differs, normalization must be made to the interval $\left[ {0,\; 1} \right]$. After sorting the absolute values of differences, the matching ranks are assigned the same value, which is the average of those ranks (zero difference values are ignored by removing them before ranking). Each rank has affixed the sign of its corresponding the signed differences. The sum of all positive and negative-signed ranks is denoted ${T^ + }$ and ${T^ - }$, respectively. The values of the last two columns enable the exact comparison of the behavior of pair of algorithms. That is a decision about the statistically significant indistinguishability of two algorithms or the superiority of one algorithm over the other. If the *p*-value is less than 0.05 and both numbers in the confidence interval are negative numbers, it means that Algorithm ${A}$ is superior to Algorithm ${B}$. Oppositely, if the numbers in the confidence interval have different signs, then neither of these two algorithms has a significant advantage over the other. Still, the larger interval length before zero compared to the interval length after zero shows that Algorithm ${A}$ is better than Algorithm ${B}$ (Derrac et al., 2011). Table 8 displays the results of the Wilcoxon signed-ranked test for a more precise performance analysis of the HDE algorithm in comparison to some of the improved methods on the CEC-2014 suite benchmark functions. The variable ${\rm MoNR}$ indicates the average rank of Algorithm $A$ relative to Algorithm $B$ in cases where Algorithm $A\;$ has a lower fitness value than Algorithm $B$. In contrast, the variable ${\rm MoPR}$ reflects the average rank in cases where Algorithm $\; B$ has a fitness value lesser than Algorithm $A$. The following two columns display the sum of the ranks. The numbers in the fifth and sixth columns indicate the number of times Algorithm $A\;$achieves superior or inferior outcomes than Algorithm $B$. Suppose the $p$-value of the seventh column is less than 0.05, and the confidence interval does not contain zero. In that case, there is a statistically significant difference between Algorithms $A\;$ and $B.$ This explanation suggests that, except for the RW-GWO algorithm, which performs identically to HDE, all other algorithms perform worse than the proposed algorithm, confirming the efficacy of this method.

**Table 8:** Nonparametric Wilcoxon signed ranked test results corresponding to the CEC-2014 test suite.

Algorithm $A$	Algorithm $B$	MoNR	MoPR	${ T^ - }$	${ T^ + }$	$ F\left( A \right)\; < F\left( B \right)$	$ F\left( A \right)\; < F\left( B \right)$	$ p$-value	Confidence interval (0.95)
HDE/current -to-best/1	MG-SCA	15.9	3.50	428	7	27	2	5.60E−06	[−7.20E+04 to −8.45E+01]
	CTLBO	15.2	8.33	381	25	25	3	5.30E−05	[−5.57E+05 to −9.20E+01]
	LJA	15.0	–	435	0	29	0	2.70E−06	[−4.90E+05 to −1.71E+02]
	m-SCA	15.0	1.0	405	1	27	1	4.47E−06	[−2.53E+06 to −5.49E+02]
	RW-GWO	14.7	13.0	235	143	16	11	2.74E−01	[−1.31E+03 to +2.82E+01]
	Cauchy- GWO	14.0	14.1	279	99	20	7	3.15E−02	[−2.38E+03 to −1.15E+00]
	ERGWO	16.5	8.43	347	59	21	7	1.08E−03	[−8.38E+03 to −2.42E+01]

### The performance of the proposed hunting mutation in the modern DE algorithms

In this section, we have investigated the performance of the proposed method as a new method in modern DE algorithms. For this purpose, we have chosen the jDE algorithm (Brest et al., 2006), one of DE’s most popular versions. Replacing the jDE's mutation with a new type of mutation, we constructed the proposed jHDE algorithm.

For comparing the performance of the proposed jHDE with the standard jDE, a set of 30 test functions was selected from the suit CEC-2014, and the optimization was run 30 times independently for each of these benchmark functions under completely similar conditions. The population for both algorithms was set to 60, the dimension of test functions was chosen to 30, and the number of iterations was set to 5,000. Therefore, each benchmark function is evaluated 300,000 times. The sign ‘ $+$’ indicates that our proposed algorithm performs better than the original jDE, the sign ‘ $-$’ indicates that it performs worse than the original jDE, and the symbol ‘ $\approx$’ demonstrates that they perform similarly.

Table 9 summarizes the simulation results of these two algorithms based on the characteristics of the mean and the standard deviation obtained from 30 independent runs. The analysis of this table leads to the conclusion that the proposed jHDE optimizer outperforms the original jDE in the mean value for 17 benchmark functions, which underlines the effectiveness of the proposed jHDE optimizer.

**Table 9:** Comparison between jDE and jHDE with some algorithms on the CEC-2014 test suite.

Function		jDE	jHDE		Function		jDE	jHDE	
F1	Unimodal	6.71E+07 1.34E+07	1.10E+07 5.18E+06	+	F17	Hybrid	2.23E+06 9.72E+05	5.84E+05 3.59E+05	+
F2		8.30E+03 6.63E+03	5.44E+03 7.57E+03	+	F18		2.81E+03 3.25E+03	1.52E+03 1.77E+03	+
F3		6.22E+01 6.39E+01	2.56E+00 2.28E+00	+	F19		8.18E+00 1.38E+00	8.24E+00 8.98E−01	≈
F4	Simple Multimodal	6.54E+01 1.53E+01	7.08E+01 2.21E−01	–	F20		2.28E+03 6.51E+02	3.95E+02 1.96E+02	+
F5		2.07E+01 4.30E−02	2.07E+01 3.30E−02	≈	F21		3.16E+05 1.02E+05	8.40E+04 4.75E+04	+
F6		2.65E+01 2.00E+00	2.42E+01 3.25E+00	+	F22		1.42E+02 5.33E+01	1.32E+02 6.06E+01	+
F7		9.09E−14 4.79E−14	7.96E−14 5.49E−14	+	F23	Composition	3.15E+02 1.72E−11	3.15E+02 5.51E−10	≈
F8		2.31E+01 4.00E+00	2.35E+01 3.73E+00	≈	F24		2.25E+02 1.89E+00	2.24E+02 1.13E+00	≈
F9		1.35E+02 1.39E+01	1.31E+02 7.61E+00	≈	F25		2.16E+02 2.97E+00	2.06E+02 9.86E−01	+
F10		4.09E+02 7.89E+01	4.62E+02 8.48E+01	–	F26		1.00E+02 4.90E−02	1.00E+02 2.88E−02	≈
F11		5.46E+03 3.69E+02	5.38E+03 1.70E+02	+	F27		6.82E+02 1.82E+02	3.22E+02 2.45E+01	+
F12		1.08E+00 2.59E−01	1.23E+00 1.11E−01	–	F28		8.60E+02 1.58E+01	8.51E+02 2.45E+01	≈
F13		3.98E−01 4.23E−02	2.95E−01 4.25E−02	+	F29		2.32E+03 7.28E+02	2.17E+03 6.29E+02	+
F14		4.08E−01 1.44E−01	3.77E−01 7.81E−02	+	F30		3.89E+03 1.23E+03	3.74E+03 1.09E+03	+
F15		1.31E+01 9.98E−01	1.34E+01 9.39E−01	≈	+			17	
					−			3	
F16		1.20E+01 2.50E−01	1.19E+01 1.99E−01	≈	≈			10	

The optimization of unimodal and hybrid test functions shows the highest superiority of the proposed optimization method. These results prove the effectiveness of the proposed method, which will probably be tested on future designed optimization algorithms.

The convergence of the proposed jHDE method can be deduced from the list of values of characteristics of jHDE and jDE optimizers for all test functions in Table 9. Still, their convergence curves can provide a better image of the convergence of both optimizers.

**Figure 4:**
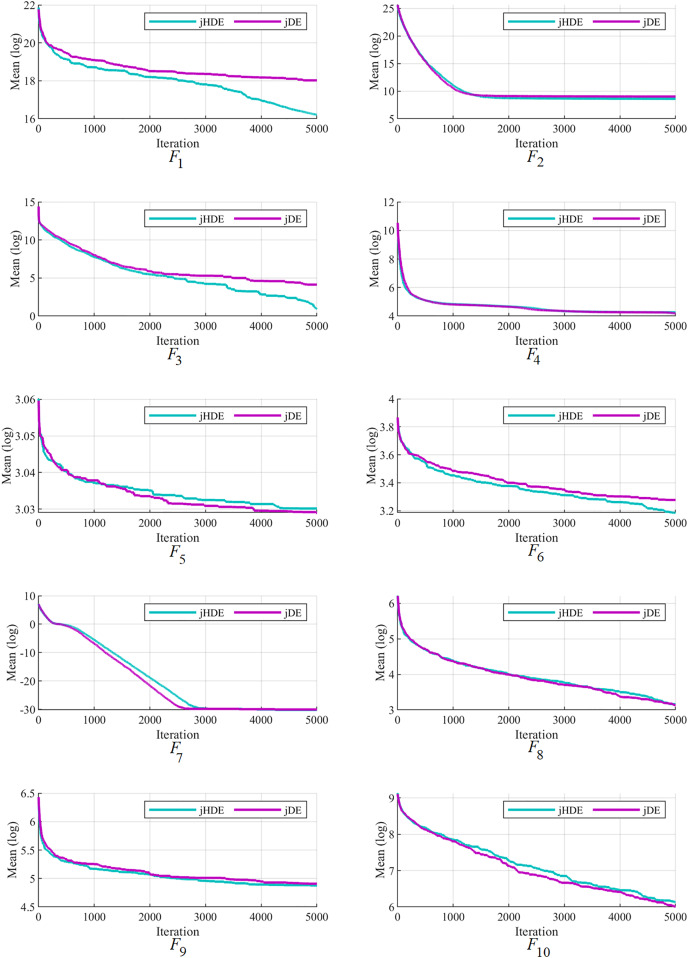
The convergence characteristics of the jDE and jHDE optimizers on F1 to F10 of the CEC-2014 test suite.

**Figure 5:**
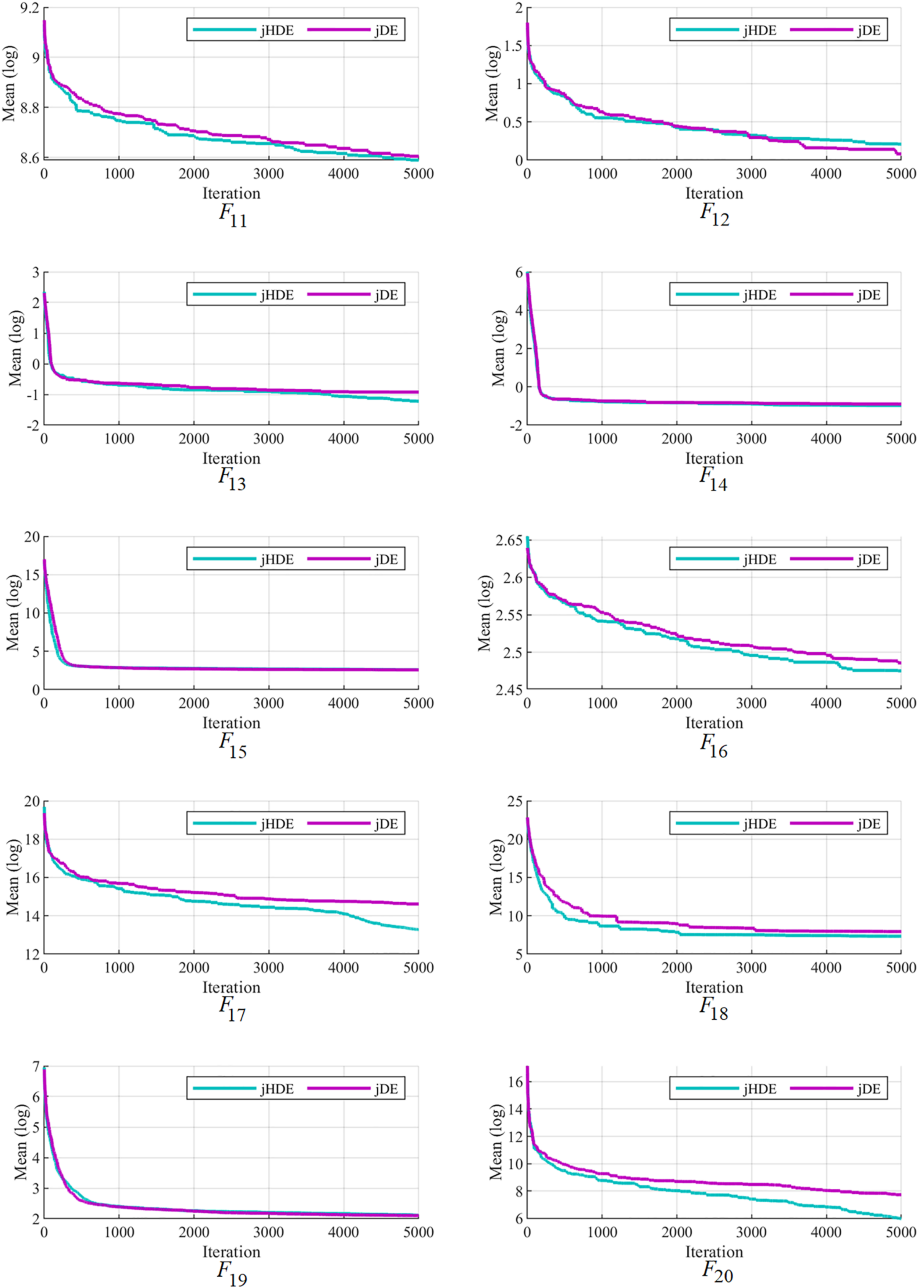
The convergence characteristics of the jDE and jHDE optimizers on F11 to F20 of the CEC-2014 test suite.

**Figure 6:**
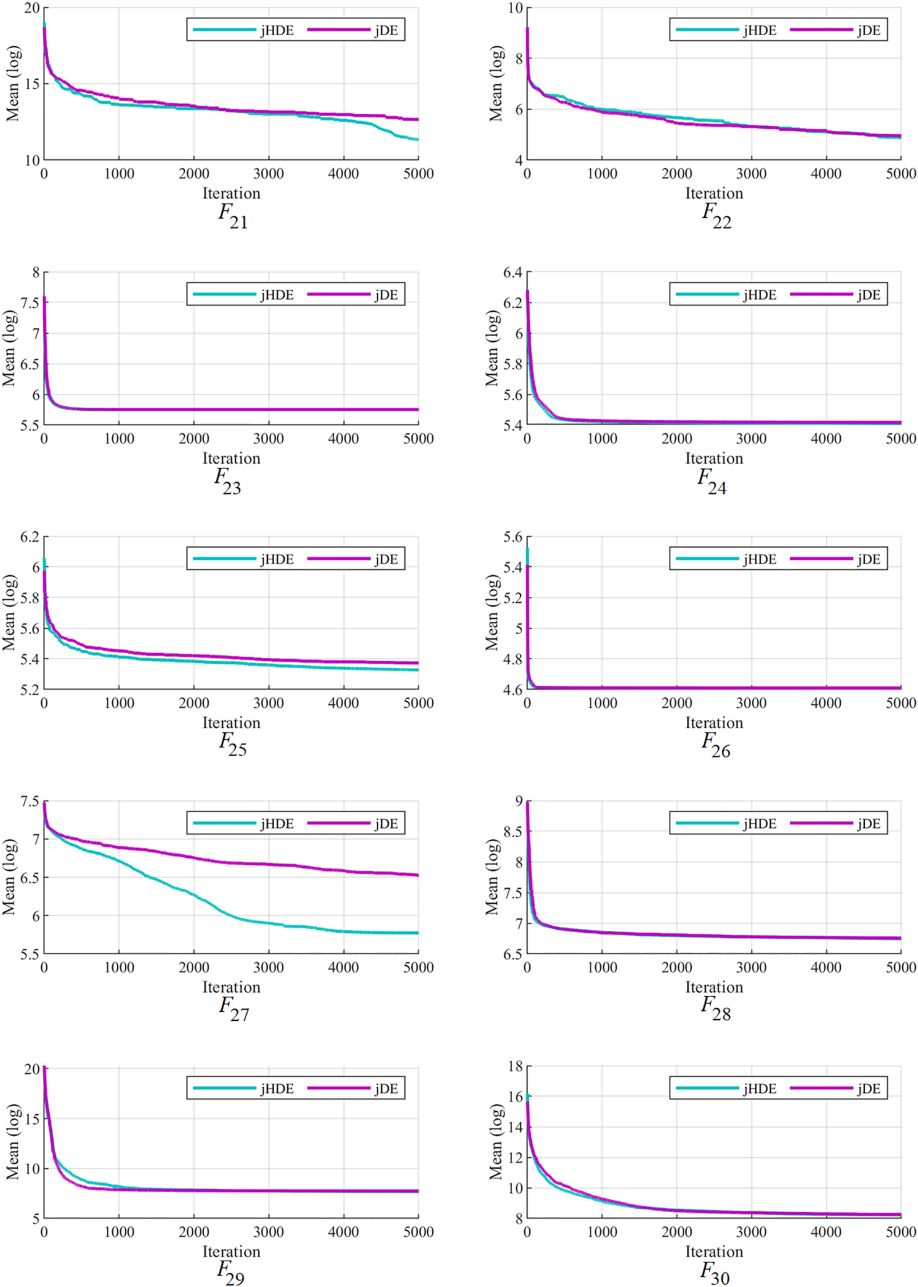
The convergence characteristics of the jDE and jHDE optimizers on F21 to F30 of the CEC-2014 test suite.

Figures 4–6 follow that the proposed jHDE has for the functions ${F_{15}}$, ${F_{19}}$, ${F_{23}}$, ${F_{24}}$, and ${F_{26}}$ convergence significantly faster than the method jDE. Thus, it is clear from these curves jHDE has a good convergence speed for most functions compared with the original jDE optimizer, which verifies the effectiveness of implementing the new type of hunting mutation in jDE.

## Conclusion

A novel meta-heuristic algorithm named Hunting Differential Evolution (HDE) was proposed by applying the Gray Wolf Optimizer (GWO) to the Differential Evolution (DE) to increase the features of the DE algorithm, such as the convergence rate. The results obtained for problems from the CEC-2014 and the CEC-2019 suites demonstrate that the proposed hunting operator was prosperous with DE algorithms. Hence, this operator can be applied to different and advanced types of other DE algorithms to improve their performances. Furthermore, the implementation of HDE algorithms to various problems from the CEC-2014 and CEC-2019 suites evidenced the strength of proposed “HDE/current-to-best/1”, “HDE/rand-to-best/2”, “HDE/rand/1”, and “HDE/best/2” algorithms for solving various engineering problems. Comparing HDE’s convergence characteristics with DE and GWO confirms that the proposed method has significantly increased convergence speed and accurately explores the feasible search space. The GWO’s features enable the population to swiftly converge towards the best solution, while the powerful DE features facilitate changes to the best solution. Combining these two features ensures continuous and rapid improvement of HDE, making it an effective optimization method. The successful performance of jHDE also indicates the potential for further improvement of HDE. Future work may include addressing the further improvement of HDE, exploring adaptive adjustment of algorithm parameters ( $CR$ and ${H_m}$) as the main drawback of the proposed algorithm, and solving engineering optimization problems.

## Supplemental Information

10.7717/peerj-cs.1420/supp-1 Supplemental Information 1 Full program code and data.

## Additional Information and Declarations

### Data Availability

The following information was supplied regarding data availability: The raw data and code are available in the Supplemental Files.
